# Impact of the Warhead of Dipeptidyl Keto Michael Acceptors on the Inhibition Mechanism of Cysteine Protease Cathepsin L

**DOI:** 10.1021/acscatal.3c02748

**Published:** 2023-10-03

**Authors:** Adrián Fernández-de-la-Pradilla, Santiago Royo, Tanja Schirmeister, Fabian Barthels, Katarzyna Świderek, Florenci V. González, Vicent Moliner

**Affiliations:** † BioComp Group, Institute of Advanced Materials (INAM), Universitat Jaume I, 12071 Castelló, Spain; ‡ Departament de Química Inorgànica i Orgànica, Universitat Jaume I, 12071 Castelló, Spain; § Institute of Pharmaceutical and Biomedical Sciences, Johannes Gutenberg-Universität, 55128 Mainz, Germany

**Keywords:** cathepsin L, inhibitor, QM/MM, MD, Michael acceptor

## Abstract

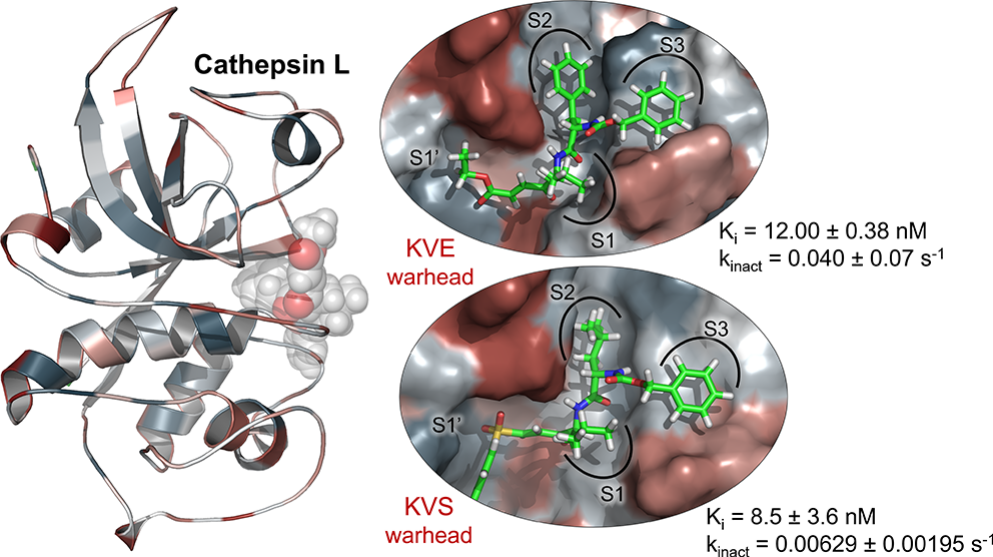

Cathepsin L (CatL) is a lysosomal cysteine protease whose activity has been related to several human pathologies. However, although preclinical trials using CatL inhibitors were promising, clinical trials have been unsuccessful up to now. We are presenting a study of two designed dipeptidyl keto Michael acceptor potential inhibitors of CatL with either a keto vinyl ester or a keto vinyl sulfone (KVS) warhead. The compounds were synthesized and experimentally assayed *in vitro*, and their inhibition molecular mechanism was explored based on molecular dynamics simulations at the density functional theory/molecular mechanics level. The results confirm that both compounds inhibit CatL in the nanomolar range and show a time-dependent inhibition. Interestingly, despite both presenting almost equivalent equilibrium constants for the reversible formation of the noncovalent enzyme/inhibitor complex, differences are observed in the chemical step corresponding to the enzyme–inhibitor covalent bond formation, results that are mirrored by the computer simulations. Theoretically determined kinetic and thermodynamic results, which are in very good agreement with the experiments, afford a detailed explanation of the relevance of the different structural features of both compounds having a significant impact on enzyme inhibition. The unprecedented binding interactions of both inhibitors in the P1′ site of CatL represent valuable information for the design of inhibitors. In particular, the peptidyl KVS can be used as a starting lead compound in the development of drugs with medical applications for the treatment of cancerous pathologies since sulfone warheads have previously shown promising cell stability compared to other functions such as carboxylic esters. Future improvements can be guided by the atomistic description of the enzyme–inhibitor interactions established along the inhibition reaction derived from computer simulations.

## 1 Introduction

Our society faces health challenges including diseases for which no remedies have been found yet or diseases with resistance to commonly employed drugs. Small molecules, inactivating key identified targets acting in the metabolic processes related to these diseases, are a valid strategy to find new chemical therapies. Cathepsin L (CatL) is a lysosomal cysteine protease belonging to the papain superfamily, which has been related to some cancerous pathologies, such as the progression of certain tumors^1,2^ or metastasis by degrading the extracellular matrix.^3,4^ CatL has become a promising target in cancer treatment as its expression is exclusively high in malignant cells, in contrast to that of other cathepsins.^5^ Inhibition of CatL might delay progression to the S phase^6^ or induce senescence^7^ or apoptosis. *In vivo* studies pointed to the combination of CatL inhibitors with regular chemotherapeutic drugs as a promising strategy to avoid drug resistance. However, clinical trials were unsuccessful with the available CatL inhibitors up to now and, consequently, it is necessary to design more efficient inhibitors.^8^ New warheads might overcome the deficiencies of the current compounds. CatL has also been identified as the major proteolytic activity to produce enkephalin neuropeptides^9^ and other neurotransmitters.^10^ Interestingly, for this catalytic role, CatL is inside neuropeptide secretory vesicles, an organelle different from lysosomes where CatL is commonly located. CatL is also related to other pathologies such as liver fibrosis, diabetes, and kidney disorders.^11−17^ Lately, the interest in developing cathepsin L inhibitors has been expanded mainly due to its possible active role in viral infection.^18^ In this regard, CatL can serve as an important modulator of the entry of Severe Acute Respiratory Syndrome Coronavirus 2 (SARS-CoV-2) into the host cell. The trimeric spike (S) protein of SARS-CoV-2 allows for the entrance of the virus into the host cell through different mechanisms. One of them is using the transmembrane protein CD147^19^ via endocytosis, but, first, part of the S protein must be cleaved. Then, membrane-bound serine (TMPRSS2)^20^ protease and CatL were found to be involved in the hydrolysis of the scissile peptide bond of the S protein.^21,22^ Compelling evidence supporting this theory is that a combination of inhibitors of both TMPRSS2 and CatL is under clinical trial as therapy against COVID-19 (the disease caused by SARS-CoV-2), at least ten of them showing favorable results in primary assays.^18^

Most successful covalent inhibitors of CatL include peptidomimetic compounds with short peptide sequences to resemble structural motifs of the natural substrate (recognition part) and a reactive part, an electrophilic moiety known as a warhead, which ends in a position favorable for the attack of the reactive cysteine (Cys25).^23,24^ This is possible thanks to the presence of the His163 residue, whose acid/basic character is modulated along the inhibition reaction by Asn187. Among the reported warheads of CatL inhibitors are electrophiles reactive toward S_N_2 substitution such as acyloxymethyl ketones^25^ and aziridines,^26^ carbonyl groups like aldehydes,^27^ as well as Michael acceptors. Most representative examples of the latter group are vinyl sulfones,^28^ vinyl sulfonates,^29^ and gallinamide A analogues.^30^

We have previously reported the keto vinyl ester (KVE) groups as a potent warhead of CatL.^31^ This warhead incorporates a ketone group at the conjugated double bond, emulating the amide carbonyl group of the substrate. This represents a structural modification of the “traditional” Michael addition inhibitors such as vinyl sulfones, in which the carbonyl group is absent.^28,32^

**Figure 1:**
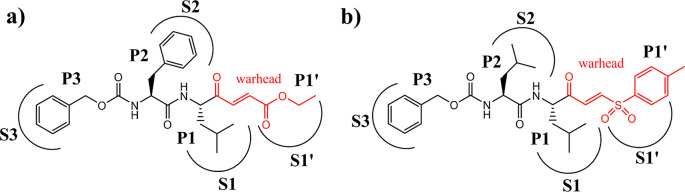
Chemical structure of proposed CatL inhibitors: (a) dipeptidyl compound **1**, with a KVE warhead and (b) dipeptidyl compound **2**, with a KVS warhead. Binding pockets occupied by different parts of the designed molecules are indicated as S1′, S1, S2, and S3.

We present a comparative study of two dipeptidyl inhibitors of CatL with different warheads (Figure 1): compound **1** containing a KVE, as previously synthesized in our group,^31^ and a new compound **2** with a keto vinyl sulfone (KVS) as a warhead that combines features of the known vinyl sulfones and the dipeptidyl KVEs. The advantages of our peptidomimetic inhibitors would be that they are expected to have great bioavailability due to their peptide nature, but at the same time, we have searched for a specific warhead that allows us to synthesize other derivatives more specific to CatL in the future. For this reason, both incorporate an extra subunit next to the reactive moiety. It is expected that the presence of the ketone group in compound **2** will interact favorably with the residues of the oxyanion hole (the backbone of Cys25 and lateral chain of Gln19), enhancing the binding affinity and thus the total activity. The recognition part of both inhibitors is made of three hydrophobic residues to mimic the natural substrates of CatL. The P1 site is occupied by l-leucine in inhibitors **1** and **2**, while l-phenylalanine and l-leucine can be found in the P2 site of compounds **1** and **2**, respectively. Both amino acids were shown to be the best fitting residues for the S2 subsite of CatL, which appears to be key for recognition and mainly accepts long chain hydrophobic residues such as aromatic and aliphatic groups.^33^ As the P3 residue, the carbobenzoxy (CBZ) moiety is used, which is expected to interact with hydrophobic residues in the S3 site, as shown in Figure 1.

In the present study, after the synthesis and *in vitro* measurements of the inhibitory activities of compounds **1** and **2**, computational methods were employed to explore whether there is any relevant difference in the CatL inactivation process with both compounds to unravel which traits are especially relevant for their selectivity and activity. Molecular dynamics (MD) simulations with classical molecular mechanics (MM) force fields (FFs) were employed to study the interactions between the two compounds and the active site of the enzyme. Subsequently, the chemical steps of the inhibition mechanism consisting of a Michael addition were studied by generating the free energy profiles (FESs) with free energy perturbation (FEP) methods using multiscale quantum mechanics/MM (QM/MM) potentials. The most favorable features of both inhibitors were evaluated, pointing out their strengths and some flaws, which are expected to be useful for designing better inhibitors for CatL. Specially, the unprecedented binding interactions of inhibitors **1** and **2** in the S1′ site of CatL represent valuable information for the future design of new inhibitors.

## 2 Methods

### 2.1 Experimental Methods

#### 2.1.1 Experimental Procedure for the Preparation of KVE, Compound **1**

Inhibitor **1** was prepared as previously reported.^31^

#### 2.1.2 Experimental Procedure for the Preparation of KVS, Compound **2**

##### 2.1.2.1 *tert*-Butyl ((4*S*)-3-Hydroxy-6-methylhept-1-en-4-yl)carbamate **4**

**Scheme 1:**
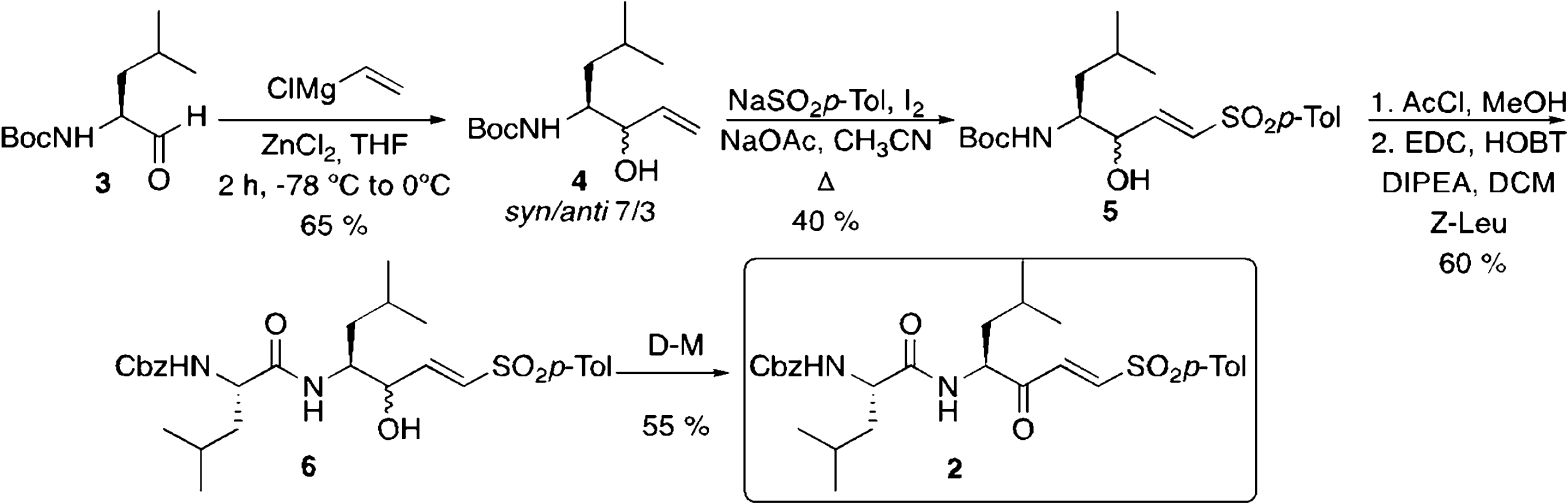
Synthesis of Inhibitor **2**

(See Scheme 1) was added to a stirred solution of aldehyde **3** (0.785 g, 3.65 mmol) in THF (18.25 mL, 5 mL/mmol) and ZnCl_2_ (0.925 g, 7.30 mmol, 2 equiv). The reaction was placed in an acetone–liquid N_2_ bath (−78 °C) and vinyl magnesium chloride (1.7 M in THF) (10.72 mL, 18.23 mmol, 5 equiv) was added under a N_2_ atmosphere. The mixture was stirred for 2 days and 22 h with warming to 0 °C. HCl 1 M (15 mL) was added, and the phases were separated. The organic phase was extracted with ethyl acetate (3 × 20 mL), and the organic layers were washed with brine, dried over MgSO_4_, and concentrated under vacuum. The reaction crude was purified by liquid chromatography (silica gel, hexane/ethyl acetate, 8:2 to 7:3) to afford a yellowish oil (0.579 g, yield = 65%).

##### 2.1.2.2 *tert*-Butyl ((4*S*,*E*)-3-Hydroxy-6-methyl-1-tosylhept-1-en-4-yl)carbamate **4**

Iodine (1.081 g, 4.26 mmol, 3 equiv) was added to a suspension mixture of **3** (345 mg, 1.42 mmol), *p*-toluenesulfinic acid sodium salt (1.563 g, 8.51 mmol, 6 equiv), and NaOAc (349 mg, 4.26 mmol, 3 equiv) in CH_3_CN (14.2 mL), and the reaction mixture was vigorously stirred at refluxing temperature for 40 h. The reaction mixture was quenched by the addition of 20 mL of saturated aqueous sodium thiosulfate (Na_2_S_2_O_3_) and basified with 20 mL of saturated aqueous sodium hydrogen carbonate. The mixture was extracted with ethyl acetate (3 × 20 mL), and the extracts were washed with water (20 mL) and brine (20 mL), dried over MgSO_4_, and concentrated under vacuum. The residue was purified by column chromatography (silica gel, hexane/ethyl acetate 8:2) to afford a yellow solid (256 mg, 40%).

##### 2.1.2.3 Benzyl ((2*S*)-1-(((4*S*,*E*)-3-Hydroxy-6-methyl-1-tosylhept-1-en-4-yl)amino)-4-methyl-1-oxopentan-2-yl)carbamate **5**

To ice-bath-cold methanol (1 mL) was added acetyl chloride (496 mL, 6.8 mmol). Then, a solution of compound **4** (174 mg, 0.45 mmol) in methanol (0.57 mL, 1.3 mL/mmol) was added, and the resulting mixture was stirred at room temperature (25 °C) for 30 min. After this time, the reaction mixture was concentrated under vacuum, and the resulting crude reaction product was submitted to the next step without any further purification. The dried crude product from the previous step was dissolved in dichloromethane (4.5 mL), and the resulting mixture was cooled with an ice-bath. Then, CBZ leucine (133 mg, 0.5 mmol), hydroxybenzotriazole (68 mg, 0.5 mmol), triethyl amine (250 μL, 1.8 mmol), and EDC (78 mg, 0.5 mmol) were sequentially added. The resulting mixture was stirred at 23 °C for 8 h, quenched with an aqueous solution of saturated ammonium chloride aqueous solution (25 mL), and extracted with dichloromethane (3 × 15 mL). The organic layers were washed with 1 M HCl solution (15 mL), then with saturated sodium hydrogen carbonate aqueous solution (15 mL), and then with brine (15 mL), dried (Na_2_SO_4_), and concentrated. The residue was purified by column chromatography (silica gel, hexane/ethyl acetate 8:2) to afford a yellow oil (98 mg, 40%).

##### 2.1.2.4 Benzyl((*S*)-4-methyl-1-(((*S*,*E*)-6-methyl-3-oxo-1-tosylhept-1-en-4-yl)amino)-1-oxopentan-2-yl)carbamate **6**

To an ice-bath-cold solution of compound **5** (98 mg, 0.18 mmol) in dichloromethane (10 mL) Dess–Martin periodinane (229 mg, 0.54 mmol) was added. The resulting mixture was stirred at room temperature (25 °C) for 3.5 h. Then, a saturated aqueous solution of Na_2_S_2_O_3_/NaHCO_3_ (10 mL) was added, and the mixture was stirred for 15 min. Then, the mixture was extracted with dichloromethane (3 × 15 mL), and the organic layers were washed with brine (15 mL), dried (Na_2_SO_4_), and concentrated. The residue was purified by column chromatography (silica gel, hexane/ethyl acetate 8:2) to afford a yellow oil (58 mg, 60%).

The dipeptidyl inhibitors **1** and **2** were fully characterized (see the Supporting Information for spectral and characterization data).

#### 2.1.3 Enzyme Assays

The inhibitory activity of compound **2** was tested against recombinant cathepsin L enzyme as reported previously.^34,35^

Enzymatic reactions were carried out with 5 μL of human CatL (Calbiochem, 1:100 dilution in enzyme buffer) in 180 μL of assay buffer (50 mM sodium acetate, pH 5.5, 5 mM EDTA, 200 mM NaCl, 0.005% Brij). 10 μL of the inhibitors (final conc.: 1000–0.78 nM) was added from DMSO stocks. Reactions were initiated by the addition of 5 μL of Cbz-Phe-Arg-AMC in DMSO (cathepsin L: 6.25 μM). Enzymatic reactions were monitored for 30 min with a Tecan Spark 10 M microplate reader (λ_ex_: 380 nm/λ_em_: 460 nm). The measurements were performed in triplicate.

### 2.2 Computational Methods

#### 2.2.1 System Setup

The X-ray structure of human CatL linked to the dipeptidyl glyoxal inhibitor PRD_000782 (PDB ID 3OF8) was taken from the Protein Data Bank as a starting point for building the models.^36^ It is important to point out that, although other high-resolution structures of cathepsin L structures have been recently deposited in the Protein Data Bank (i.e., 8C77, 8OZA, 7QKB and 7W34 IDs), the 3OF8 structure presents the advantage of having an inhibitor in the active site close to the ones proposed in the present study. In addition, comparative analysis shows no significant structural differences in the active site of the mentioned crystallized structures and the one selected in the present study (see the Results and Discussion Section), thus supporting our selection of the initial set of coordinates to set up our models. Thus, PRD_000782 was used as a template structure of both compounds **1** and **2** that were manually generated to obtain the E:I reactant complexes. Missing parameters for both warheads and the CBZ group were obtained using General Amber Force Field,^37^ and the atomic charges were computed with the AM1 method with bond charge corrections^38^ using Antechamber software^39^ (Table S1), which is available in the AmberTools package. The values of p*K*_a_ of the titratable residues were determined using the empirical program PropKa v.3.0.3.^40^ According to the results (Table S2), shifts from the standard p*K*_a_ values in solutions were identified in some residues, despite not being very dramatic. Interestingly, the p*K*_a_ of His140 and His208 are 7.09 and 6.29 respectively, suggesting that at pH 5.5, they would be positively charged, but at pH higher than 7, they would be neutral. It is also relevant that both residues are at the end of an α-helix and at the beginning of a loop, none of them too far from the active site (in a range of 10–15 Å). Thus, even though they are not part of the active site, a conformational change in the neighboring region due to a change in the protonation state of these residues could take place if pH is increased. In fact, experimental assays over CatL made by Dufour et al.^41^ confirmed that over pH 6, the activity of the enzyme is lost, in agreement with a possible conformational change of the protein that could affect the reactivity of the enzyme. Three disulfide bridges that formed between Cys22 and Cys65, Cys 56 and Cys98, and Cys156 and Cys209 were identified, and consequently, hydrogen atoms were not added to the Sγ of these residues. The charge of the system was neutralized by the addition of seven sodium cations placed in the most electrostatically favorable positions using the tleap software,^37,39^ and the whole complex was solved in a box of water molecules of 65 × 68 × 86 Å^3^ treated with TIP3P FF.^42^ The total systems contained 3273 atoms of the protein, 7 sodium ions, 8979 and 8918 water molecules, and 69 and 75 atoms of **1** and **2** inhibitors, respectively.

#### 2.2.2 MM MD Simulations

A series of minimizations of 10^5^ steps were performed on both models using the conjugate gradient algorithm, followed by conventional MD simulations with the NAMD software,^43^ using an *NVT* ensemble with an AMBER ff03 FF.^44^ First, a minimization of the newly built inhibitors (**1** and **2**) with constrained positions of atoms of the protein was performed, allowing for adapting their optimal position in the active site. Subsequently, the process was repeated for the protein with fixed positions of the atoms of inhibitors, and the final step of minimization was done without applying any constraints. Both models were heated to the temperature of experimental conditions, that is, 298 K, through a 10 ps MD using a constant increment of 0.1 K/fs. Then, 100 ps *NPT* MD at 298 K using a Langevin piston^45^ was carried out. From the last frame of this trajectory, 50 ns *NVT* MD was carried out constraining the distance between Sγ of Cys25 and the Cα of the double bond of inhibitor warheads. Finally, 1 μs free *NVT* MD simulations were carried out to equilibrate the systems. For all these simulations, periodic boundary conditions were applied. The selected time step was 1 fs, and the particle mesh Ewald algorithm^46^ was employed to account for the nonbonding interactions, with a smooth switching function at a distance from 14.5 to 16 Å. The root-mean-square deviation (rmsd) along the dynamics was computed in order to verify that both systems are genuinely equilibrated (Figure S1). For this, we used the cpptraj program,^47^ as provided with AmberTools.

#### 2.2.3 QM/MM Free Energy Calculations

**Figure 2:**
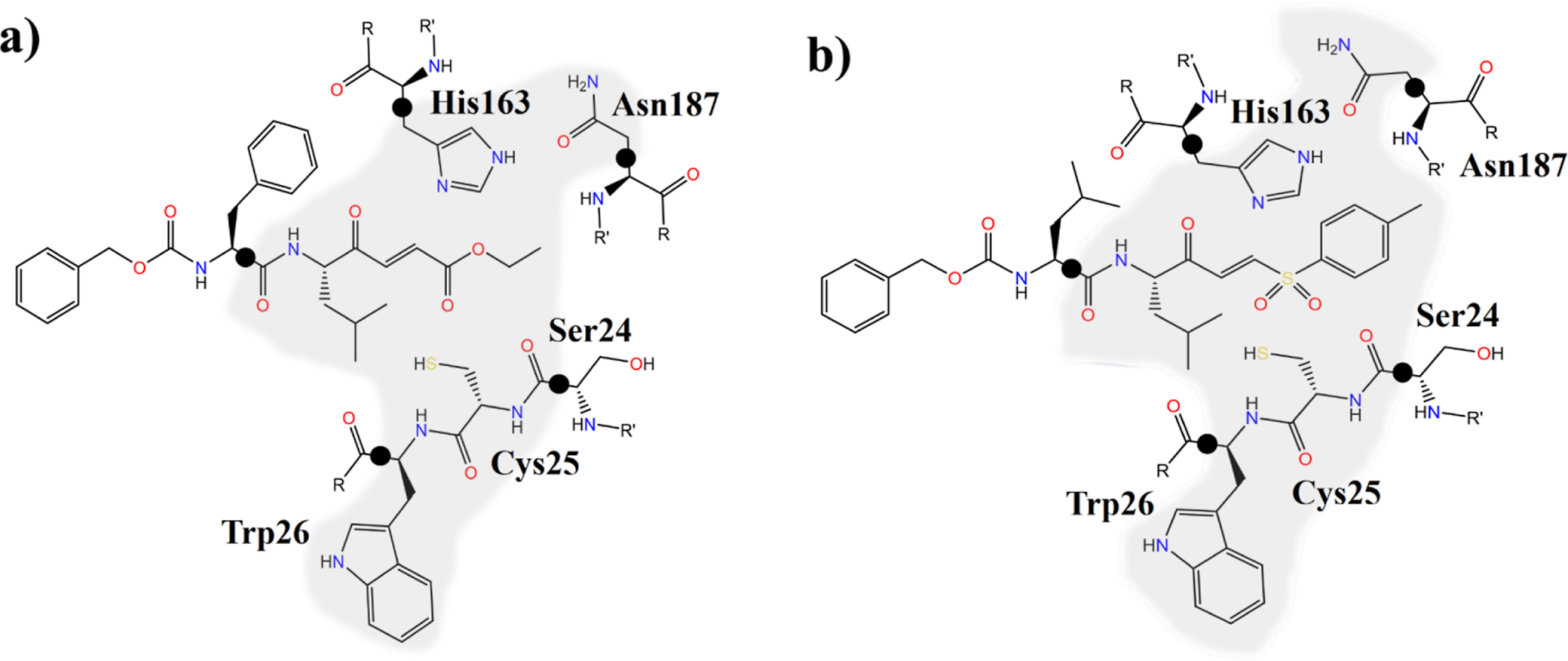
Schematic representation of the active site of CatL with (a) compound **1** and (b) compound **2**. The QM regions are indicated in gray. The black dots indicate the positions of the quantum link atoms.

For the study of reaction mechanisms, we have chosen an additive hybrid QM/MM^48^ approach to construct the overall Hamiltonian, using the last snapshot of the previous equilibration MD simulations as the starting point. A comparative analysis between the averaged geometrical values of key distances in the equilibrated structures obtained along the long MM MD simulations of the noncovalent reactant complex and the selected final structure employed for the QM/MM calculations confirms that the latter represents the most populated geometrical configurations of the reactant state (see Figure S2). The QM region, as illustrated in Figure 2, was defined by selecting part of the inhibitors, residues of the catalytic triad formed by Cys25, His163, and Gln187, as well as Trp26 and the carbonyl group of Ser24 neighboring the reactive cysteine. Five quantum link atoms were inserted for treating the QM/MM frontier, leading to QM regions of 89 and 96 atoms for **1** and **2**, respectively.

In order to study the Michael addition mechanism, the QM subsets were treated with the M06-2X^48^ density functional with the 6-31+g(d,p) basis set, while the rest of the protein and the solvent water molecules were described by AMBER and TIP3P FFs, respectively, as implemented in the fDynamo^49,50^ library. QM/MM potential energy surfaces (PESs) were generated using Gaussian09^51^ ver. D.01 combined with fDynamo as routinely used in previous studies in our laboratory.^52−54^ All the stationary points were optimized at the DFT/MM level, including transition states (TSs), using Baker’s algorithm.^55^ For all TSs, the Hessian was computed and diagonalized to confirm the existence of a single negative eigenvalue. The convergence of these states was achieved when a 1.2 kJ mol^–1^ Å^–1^ energy gradient was reached. The intrinsic reaction coordinate (IRC)^56,57^ method was applied to the optimized TSs to trace down the minimum energy paths heading to the closest minima. The final structures obtained from IRC were characterized using the same optimization procedure as that described above.

The FEP method was applied to generate the free-energy profiles of each step of the reaction.^58,59^ This consists of sampling the MM region through the path traced by the previously calculated IRC at the M06-2X/MM level of theory. The change in the MM part during this exploration provokes a polarization in the QM wave function, which allows the exploration of each step at this level of theory. Thus, the MM environment has a profound impact on the QM/MM potential energy profile. Furthermore, since the QM area variation comes from the IRC connecting common states in each step (ionic pair state in our case), it can be said that we are following a realistic reaction coordinate.

Structures from the IRC were extracted and characterized by defining an *s* coordinate as defined in eq 1
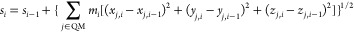
1Here, *m*_*i*_ is the total mass of the QM region and *x*_*j*,*i*_,*y*_*j*,*i*_ and *z*_*j*,*i*_ are the coordinates of the atom *j* of structure *i* of the QM subset.

According to the *s* coordinate definition, the change of the free energy is computed following eq 2
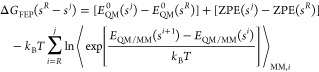
2where *E*_QM_^0^ is the QM gas phase energy computed at the M06-2X level, ZPE is the minimum energy that the system can have in the vacuum known as the zero-point energy, *k*_B_ is the Boltzmann constant, and *T* is the temperature of the system. The last term of the equation accounts for the QM/MM contribution to the free energy as an average of the QM/MM interaction energy difference between one s state and the next one, over all configurations of the atoms in MM explored during the MD simulations. In this particular case, we explored QM/MM MD during 20 ps for each window extracted from the IRC path at 298 K using the *NVT* ensemble and fixing positions of the atoms of the QM subset during the simulation. For the first step, which corresponds to the proton transfer, 37 and 26 windows were used in the case of compound **1** and compound **2**, respectively. In the second step, which describes the Michael addition process, 83 and 103 structures for compound **1** and compound **2** inhibitors were selected.

## 3 Results and Discussion

As previously reported,^31^ the synthesis of compound **1** was accomplished in a three-step sequence. The KVE warhead was made by a Horner–Wadsworth–Emmons reaction between the corresponding dipeptidyl phosphonate and ethyl glyoxal. However, for the preparation of compound **2**, a different synthetic strategy was employed. First, *tert*-butoxycarbonyl leucinal **3** was treated with vinyl magnesium chloride affording a diastereomeric mixture of allyl alcohols **4**. The carbon–carbon double bond of compound **4** was then converted to vinyl sulfone **5** by reaction with sodium *para*-toluene sulfinate and iodine.^60^ Then, compound **5** was transformed into dipeptidyl compound **6** by removal of the *tert*-butyl carbamate group upon treatment with acetyl chloride and methanol followed by peptide coupling with *N*-carbobenzoxy leucine under standard conditions. Dess–Martin oxidation of alcohol **6** afforded inhibitor **2** (Scheme 1).

**Figure 3:**
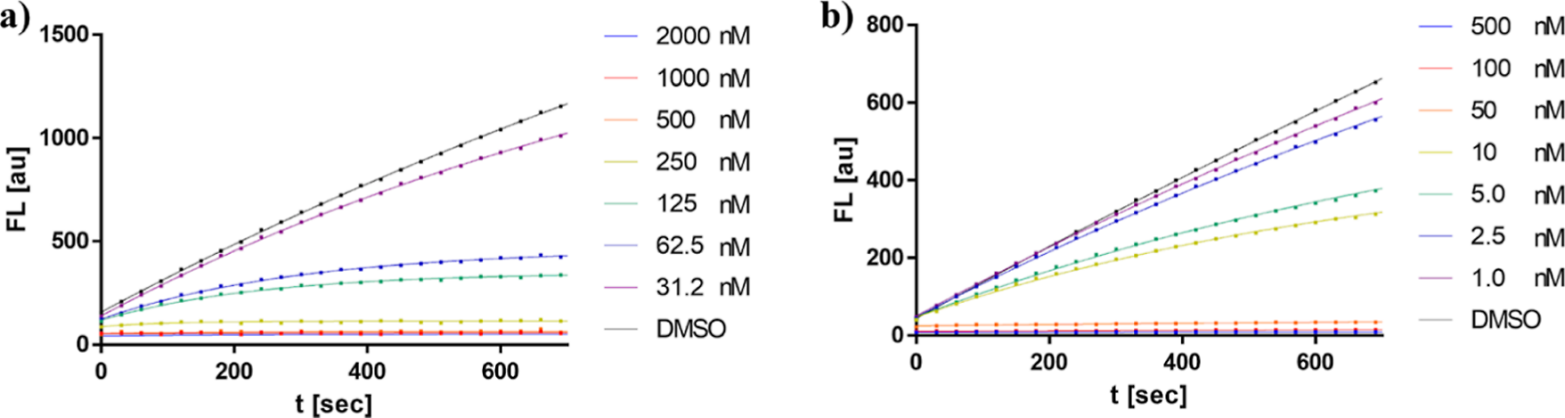
Fluorometric CatL with varying concentrations of (a) compound **1** and (b) compound **2**.

The desired inhibitors, **1** and **2**, were obtained with high yields and purities (see the Supporting Information for details) and were submitted to *in vitro* testing with the recombinant cysteine protease CatL (see Table 1). The progress curves of substrate hydrolysis by CatL with varying concentrations of compounds **1** and **2** show the time-dependent mode of inhibition (Figure 3).

**Table 1:** Experimental Data Obtained from the *In Vitro* Assays of CatL with **1** and **2**

compound	*K*_*i*_ (nM)	*k*_inac_ (s^–1^)	*k*_inac_/*K*_*i*_ (M^–1^ s^–1^)
**1**	12.00 ± 0.38^a^	0.040 ± 0.007^a^	3.300.000 ± 528.000^a^
**2**	8.5 ± 3.6	0.00629 ± 0.00195	740.000 ± 110.000

a Data from Reference (30).

**Scheme 2:**
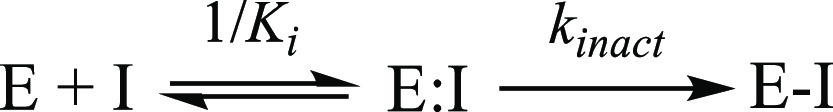
Inhibition Reaction

The kinetic constants of the inhibition steps, the equilibrium constant (1/*K*_*i*_) for the formation of the E:I reversible complex, and kinetic constant *k*_inact_ for the formation of the final E–I covalent complex (Scheme 2) were then determined (Table 1).

In fact, when the *in vitro* inhibition assays were performed, it was shown that the two compounds are well recognized by the active site, and they show a notable reactivity against CatL (Table 1). The constant of the first reversible step (*K*_*i*_) was similar for both inhibitors but the kinetic constant of the second step was 1 order of magnitude higher for compound **1** than for compound **2** (Table 1). It is important to point out that while *K*_*i*_ depends on the noncovalent interactions formed between the recognition part of the inhibitor, *k*_inact_ is influenced by intrinsic compound reactivity, which mainly, but not exclusively, depends on the warhead. The equilibrium constant values indicate that the formation of the reversible E/I complex is highly favored for both inhibitors (similar *K*_*i*_ in the nanomolar range) while KVE **1** has higher kinetics for the formation of the irreversible covalent E–I complex as compared to KVS **2**.

Despite values reported in Table 1 suggesting compound **1** to be slightly more reactive than compound **2**, keeping in mind future possible developments of drugs based on these compounds, the ester group present in inhibitor **1** is potentially hydrolyzable, lowering the stability in cell media.^61^ In this sense, the sulfone group present in inhibitor **2** represents, a priori, a better alternative.

### 3.1 Computational Study of the Noncovalent E:I Reactant Complex

**Figure 4:**
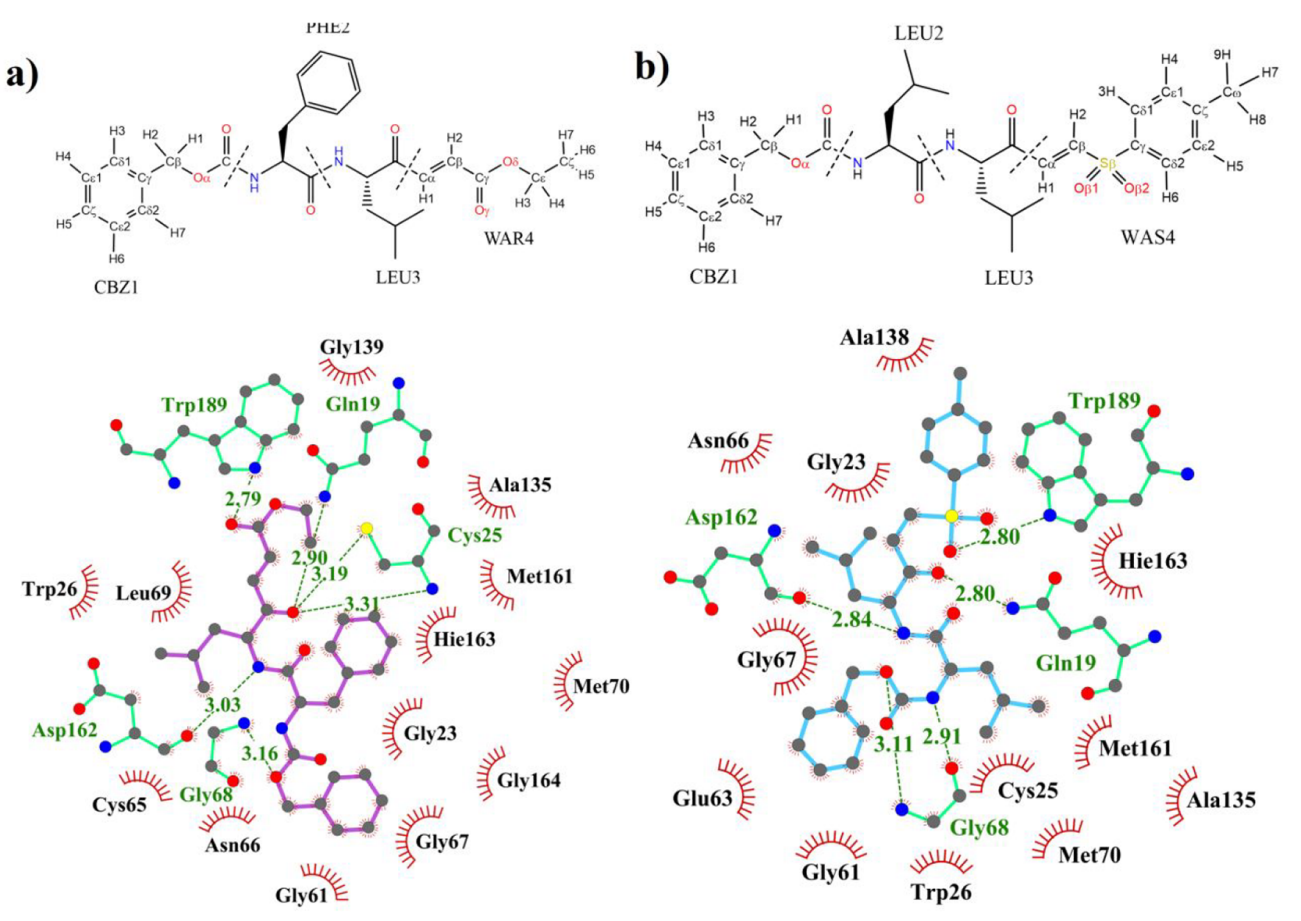
Numbering of the atoms of (a) compound **1** and (b) compound **2**; schematic representation of the main protein/inhibitors interactions (in dashed lines) generated with the LigPlot+ program.^62^

The analysis of the evolution of the two systems observed during 1 μs of unconstrained MD simulations at the noncovalent E:I reactant complex allowed us to gather information about the binding of the two inhibitors to the active site of CatL (Figure 4). The time evolutions of the rmsd’s of the protein backbone and the full protein were evaluated and compared (Figure S1). The results show that, in both cases, the protein backbone and the lateral chains are equilibrated after the first 100 ns of MD simulations. When focusing on the behavior of the inhibitors inside the binding pocket, it was noticed that the warheads of both compounds appear to be significantly flexible, in contrast to the P2 and P3 residues, for which oscillations are dramatically smaller (Figure S3). However, and despite minor differences, both inhibitors can be considered as equilibrated after 500 ns, with the warhead perfectly adjusted to the S1′ pocket and correctly orienting the double bond for the attack of Cys25 (Figure S4). We hypothesize that the fact that the sulfone group contains a bulky *p*-tolyl group can be responsible for the KVS warhead being slightly more mobile (see Figure S4). The KVE, in contrast, has a considerably smaller ester group and an ethyl group that can adapt to any nearby hydrophobic cavity, thus facilitating the orientation that favors the Sγ^C25^–Cα^WAR4-**1**^ interaction.

**Figure 5:**
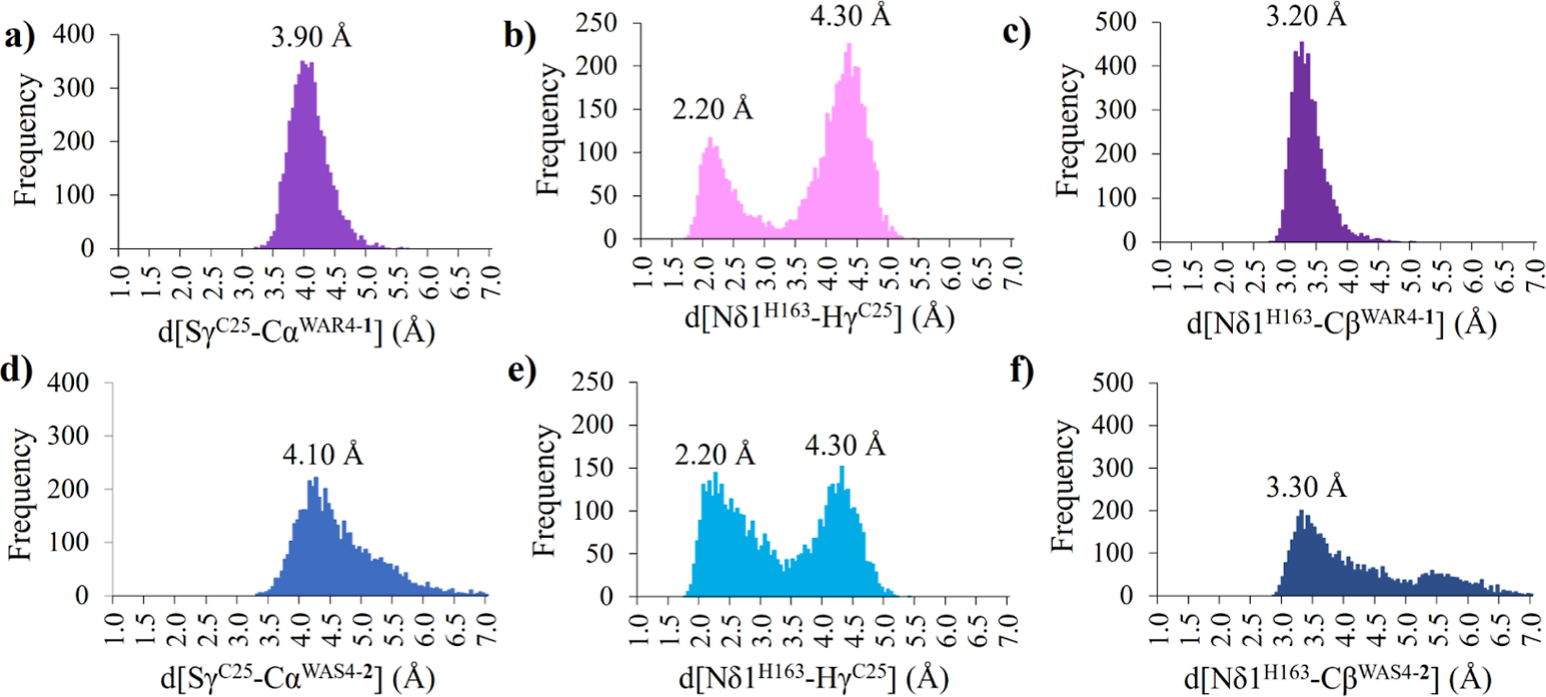
Distribution of distances along the last 500 ns (using 5000 structures) of MD simulations for compound **1** (a–c) and compound **2** (d–f).

Regarding the rest of the key distances in the E:I complex, the results support the prediction of the stable, and correctly oriented for the reaction, character of the noncovalent complexes (Figure S5). This can be deduced by observing the evolution of Nδ1^H163^–Hγ^C25^ distance, which defines whether the Cys25 can be activated by His163, the Sγ^C25^–Cα^WAR4-**1**^ and Sγ^C25^–Cα^WAS4-**2**^ distances assuring the cysteine attack to the double bond of compounds **1** and **2**, respectively, and the Nδ1^H163^–Cβ^WAR4-**1**^ or Nδ1^H163^–Cβ^WAS4-**2**^ distances, which determine the possible proton transfer from His163 to the inhibitor once the enzyme–inhibitor covalent bond was formed (as discussed in the next section). The population analysis of these key distances, computed with the structures generated in the last 500 ns MD simulations, shows that the Sγ^C25^–Cα distances are around 3.9 and 4.1 Å for compounds **1** and **2**, respectively, in the most frequently appearing structures (Figure 5a,d). The Nδ1^H163^–Cβ distances are 3.2 and 3.3 Å for compounds **1** and **2**, respectively (Figure 5c,f), despite larger values also being populated in the latter. Regarding the Nδ1^H163^–Hγ^C25^ distance (Figure 5b,e), the distribution is almost the same in both compounds, now presenting two different equally distributed conformations; a reactive one with a distance of 2.2 Å and a nonreactive conformation with a distance of 4.3 Å. However, the nonreactive conformations, as defined by these two distances, appear at a shorter time of the dynamics (especially in the case of compound **2**), while the more reactive ones correspond to longer times when the systems can be considered as fully equilibrated (Figure S5).

**Figure 6:**
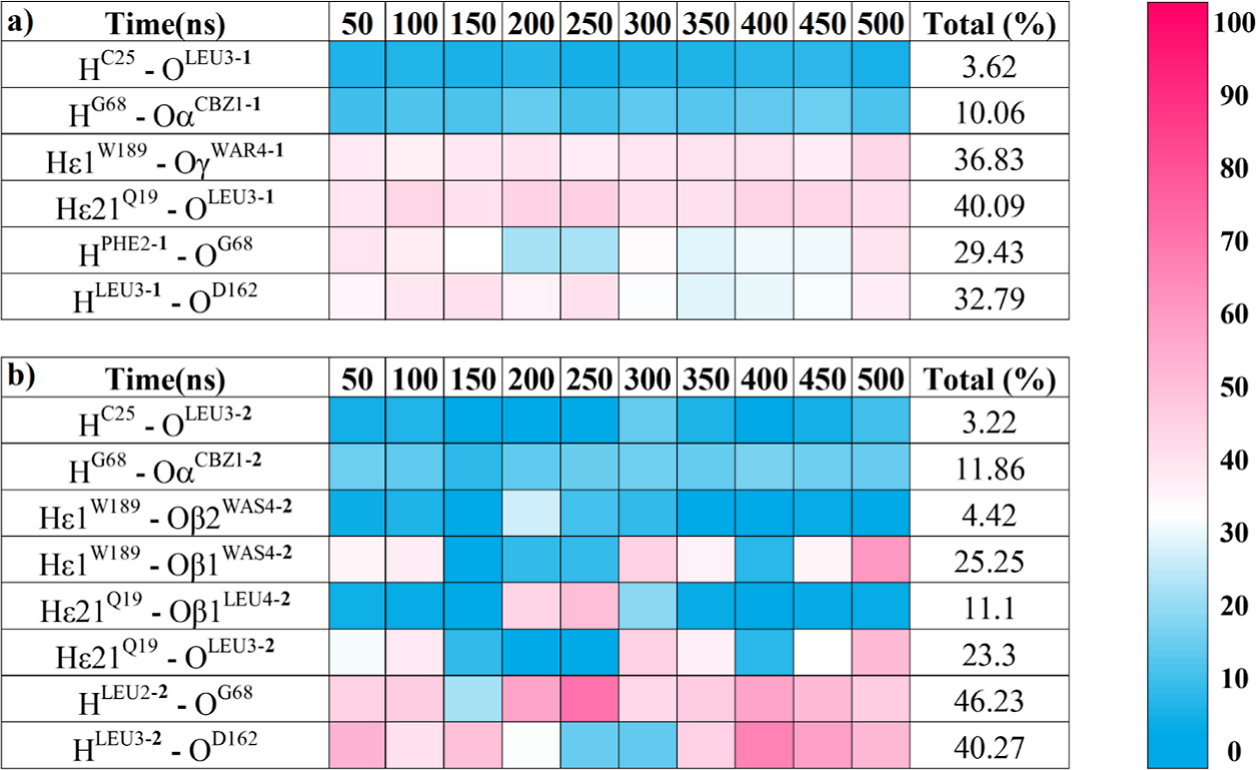
Heat plot of protein/inhibitor hydrogen bond contacts divided into blocks of 50 ns in compound **1** (a) and compound **2** (b). Values were obtained by defining the default cutoff distance between the hydrogen donor and its acceptor to 3.0 Å and the angle formed between the donor, hydrogen, and acceptor to 135°.^63^

Analysis of the population of the key hydrogen bond interactions established in the active site along the MD simulations (Figure 6 and S6) provides additional information on relevant short-distance contacts that contribute to stabilizing the inhibitors in the active site of CatL.

Compound **1** shows four H-contacts identified as the most preserved ones during the simulations, formed between the indole of Trp189 and the carbonyl of the ester group (Nε1^W189^–Hε1^W189^···Oγ^WAR4-**1**^), the carbonyl next to the reactive double bond and the side chain of Gln19, one of the residues of the oxyanion hole (Nε2^Q19^–Hε21^Q19^···O^LEU3-**1**^), as well as the Gly68 and Asp162 carbonyl groups of the protein backbone with the N–H of the linker’s phenylalanine (N^PHE2-**1**^–H^PHE2-**1**^···O^G68^) and leucine (N^LEU3-**1**^–H^LEU3-**1**^···O^D162^), respectively. Regarding compound **2**, the pattern of interactions is similar to the one identified in compound **1**. However, despite this similarity, some differences are of relevance, which must be taken into account for future refinements of the inhibitors. Thus, N^LEU2-**2**^–H^LEU2-**2**^···O^G68^ and N^LEU3-**2**^–H^LEU3-**2**^···O^D162^ show an increase of 16.8 and 7.5% in the total average of preservation with respect to the corresponding ones on compound **1**. It seems especially remarkable that the change of a phenylalanine to a leucine has, at least, increased the strength of binding of the core of the inhibitor. On the other hand, interactions with the oxyanion hole residue Gln19 have decreased considerably (16.8%), as well as the interactions of the Oβ1 oxygen of the sulfone with Trp189, which have decreased 11.3%. This is followed by the appearance of new contacts with both Gln19 and Trp189 during the dynamics. These contacts come on stage when the sulfone adopts a conformation in which Oβ1 interacts with Gln19, provoking nonreactive conformations (large Sγ^C25^–Cα^WAR4-**1**^ distance). In contrast, when Oβ1 is interacting favorably with Trp189, the *p*-tolyl group is mainly pointing out to the solvent, favoring the Sγ^C25^–Cα^WAR4-**1**^ distance that corresponds to reactive conformations (Figure S4d).

**Figure 7:**
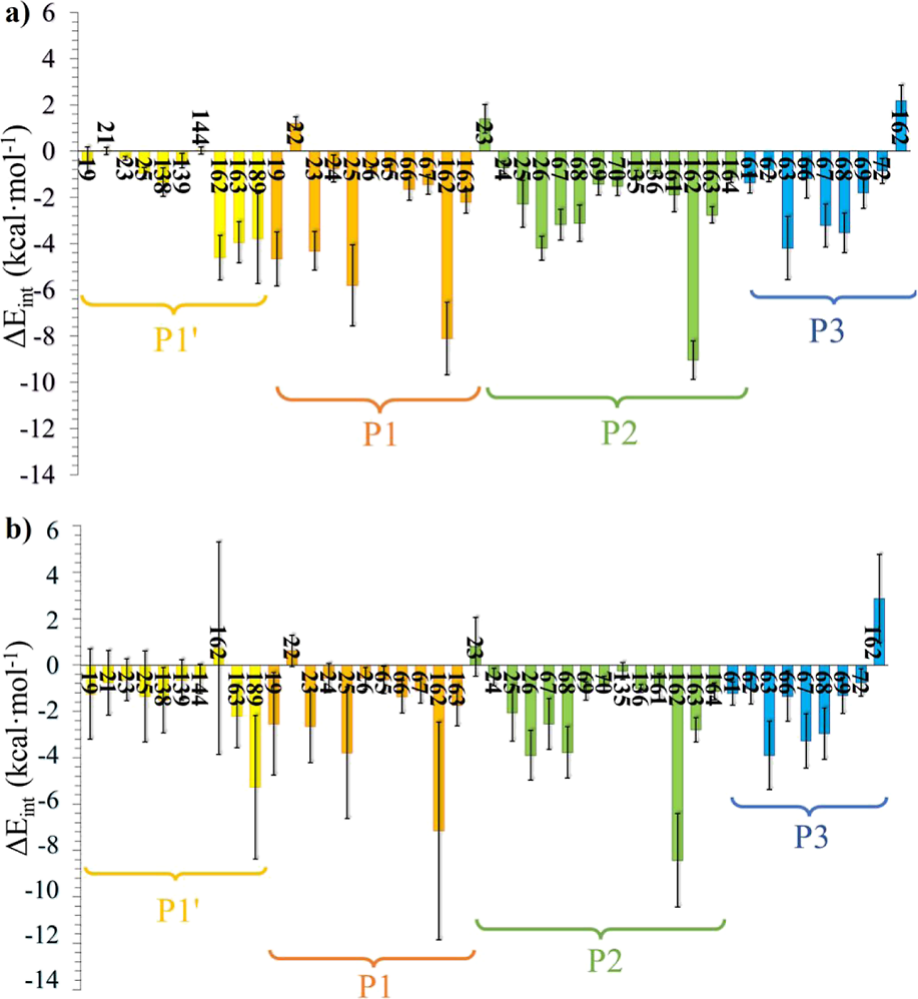
Average interaction energy (*E*_elec_ + *E*_LJ_) computed between each P site of (a) compound **1** and (b) compound **2** and the residues of CatL based on 5000 snapshots generated during the last 500 ns of the MD simulation.

**Figure 8:**
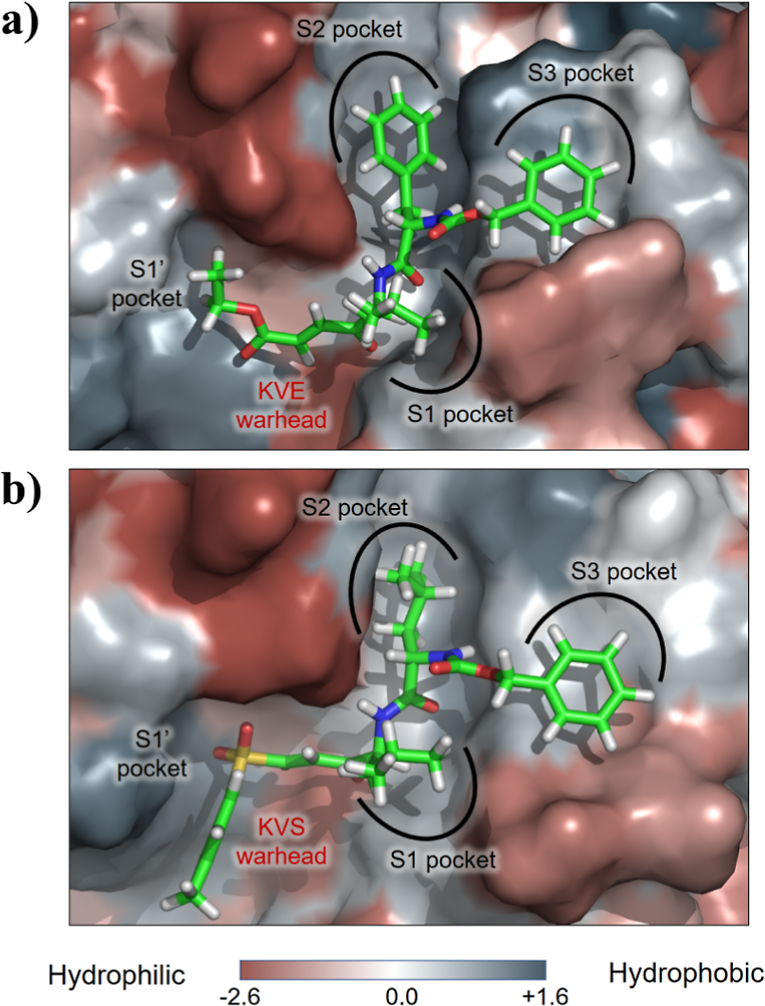
Position of compounds (a) **1** and (b) **2** in the binding pocket of CatL. The hydrophobicity map of the binding pocket of the CatL generated using the Eisenberg scale.^64^

The average interactions (electrostatic plus van der Waals) between the inhibitors and the protein, decomposed by residue, are shown in Figure 7, while a graphical representation of the most representative poses of the two inhibitors in the active site of CatL is shown in Figure 8. The comparative analysis between both systems allows a better understanding of the effect of the warhead and the recognition part in the complete pattern of interactions responsible for the noncovalent E:I complex stability.

Interestingly, despite the difference in the residue on the P2 site, l-phenylalanine in compound **1** and l-leucine in **2**, the P2 and P3 moieties show almost quantitative equivalent interactions for both compounds. Among all of these, the destabilizing electrostatic effect of the negatively charged carboxylate group of Asp162 on the P3 site is remarkable. On the other hand, the attractive interactions with Gly68 through an H-bond, Gly67 and Asp63 were found, which overall compensate for the unfavorable effect of Asp162. Moreover, the Cbz group was found to have purely hydrophobic interactions with Leu69 and Tyr72. In the P2 position, even though different residues are inserted in **1** and **2**, these have very similar chemical features, with the particularity that the Phe2 in **1** has an aromatic ring which is more bulky and rigid than the isobutyl chain in the Leu2 of **2**. From an energetic point of view, we barely see any difference in the interactions formed between protein and these two residues, apart from the bigger variance for Leu2 (**2**) than for Phe2 (**1**), a fact that can be explained by the higher flexibility of the isobutyl group compared to the benzyl group. Therefore, the most relevant interactions are those formed with Gly68 through a hydrogen bond, with Gly67 through electrostatic contacts and with Asp162 when the carboxylate points toward the amide bond of both inhibitors. The side chain in both cases is stuck inside the S2 pocket, where many predominantly van der Waals interactions are appearing such as those with Trp26, Leu69, Met70, His163, or Gly164. These interactions together with the wrapping effect of this pocket provoke the anchoring of P2 groups of both inhibitors in S2. Besides, there is a small repulsive force between the carbonyl of the P2 residue and the carbonyl of Gly23 since they are oriented to one another.

The interactions of the P1 side chain in the S1pocket in both **1** and **2** are qualitatively equivalent. In both cases, the most important interactions are those established with the oxyanion hole, which comprises the backbone of Cys25, that is, N–H group and the side chain of Gln19, which forms moderate/weak hydrogen bonds with the carbonyl of P1, as discussed above, and also with Asp162 through another hydrogen bond. Regarding the side chain of P1, it interacts only with Gly23, which is due to the shallowness of the S1 pocket. Interestingly, analysis of the evolution of interaction energies along the MD simulations between Asp162 and P1 (Figure S7) shows that while the interaction with **1** has always a stabilizing effect, higher fluctuations between stabilizing and nonstabilizing interactions are observed in **2**, in agreement with the higher mobility of the inhibitor discussed above.

Finally, the designed warheads have in both cases a subunit able to interact with the residues of the S1′ pocket. The most relevant interactions for **1** are predominantly electrostatic such as with Asp162, His163, and the hydrogen bond interaction formed between the carbonyl of the ester and the indole group Trp189. There are also other minor interactions between the ethyl chain of the warhead and the S1′ residues such as Ala138 and Gly139. On the other hand, **2** interacts similarly with His163 and Trp189, but there is a repulsive interaction with Asp162. Again, the variance in the energies is much higher in **2** than in **1**, which can be attributed to the fact that while the ester interacts favorably with the carboxylate of Asp162, the positioning of the sulfone is provoking that in a high population of structures, the Oβ2 is pointing toward the carboxylate of this residue, generating a repulsive force that makes the warhead more unstable (Figure S7). Furthermore, although the *p*-tolyl group of **2** can be too bulky to fit in the S1′ pocket, several conformers in which it interacts favorably with residues located further from the active site of the protein have been found, as shown in Figure 8.

### 3.2 Chemical Steps of the CatL Inhibition Process

The recognition step was just the prelude to the chemical transformations that both tested compounds experience in CatL covalent inhibition. The stabilization of the final product of this process determines the character of inhibition related to its reversibility. Therefore, a study of the chemical reaction mechanisms and their energetics can shed light on aspects that can be taken into consideration during the design of improved enzyme inhibitors. In the case of cysteine proteases, and CatL in particular, the study of the mechanism is additionally relevant because it can provide information on the most stable protonation state of active site Cys25 and His163 catalytic dyad in the reactant state, that is, the noncovalent E:I complex. This topic has been extendedly argued, and it has been concluded that one cannot be completely certain of the protonation state unless there is an available high-resolution NMR resolved crystal structure of the protein under study.^65^ In the case of CatL, this information is not available, so we used the results from calculations to determine the protonation state.

Based on the geometries of the E:I complex obtained from the MD simulations, different reaction mechanisms can be proposed. First, DFT/MM PESs were generated to explore the possibility of a concerted initial step involving the proton transfer from Cys25 to His163, concomitant with the attack of Sγ^C25^ to the double bond of the inhibitors warhead. According to our results (Figure S8), it was concluded that the first step was a stepwise process, in which the transfer of the proton precedes a nucleophilic attack. Consequently, a first step involving the proton (Hγ^C25^) transfer from Cys25 to His163 resulting in the activated ionic pair (E:I^(±)^) was first explored. The free energy barrier for this step is 3.4 kcal mol^–1^ higher for **1** than that for **2** (see Figure S9). A priori, the difference in the stability of the ion pair and the associated barrier for proton transfer, **TS1**, between the two inhibitors should not be affected by the inhibitor since this first chemical step is associated with a proton transfer between two protein active site residues. However, different geometrical and electronic effects can be imposed by the inhibitors in the active site and, consequently, slightly different energetics can be obtained. In fact, this difference agrees, to some extent, with the geometrical differences in the E:I, where the distance between Cys25 and His163 is larger in **1** than in **2** (2.5 vs 2.3 Å). Analysis of charges (see Tables S5 and S6) reveals no significant differences between both reactions in the first step, apart from the fact that the Sγ–Hγ bond of Cys25 appears to be slightly more polarized in the E:I bond of compound **1** than in compound **2**. However, the evolution of the charge distribution from E:I to **TS1** is equivalent in both reactions.

**Figure 9:**
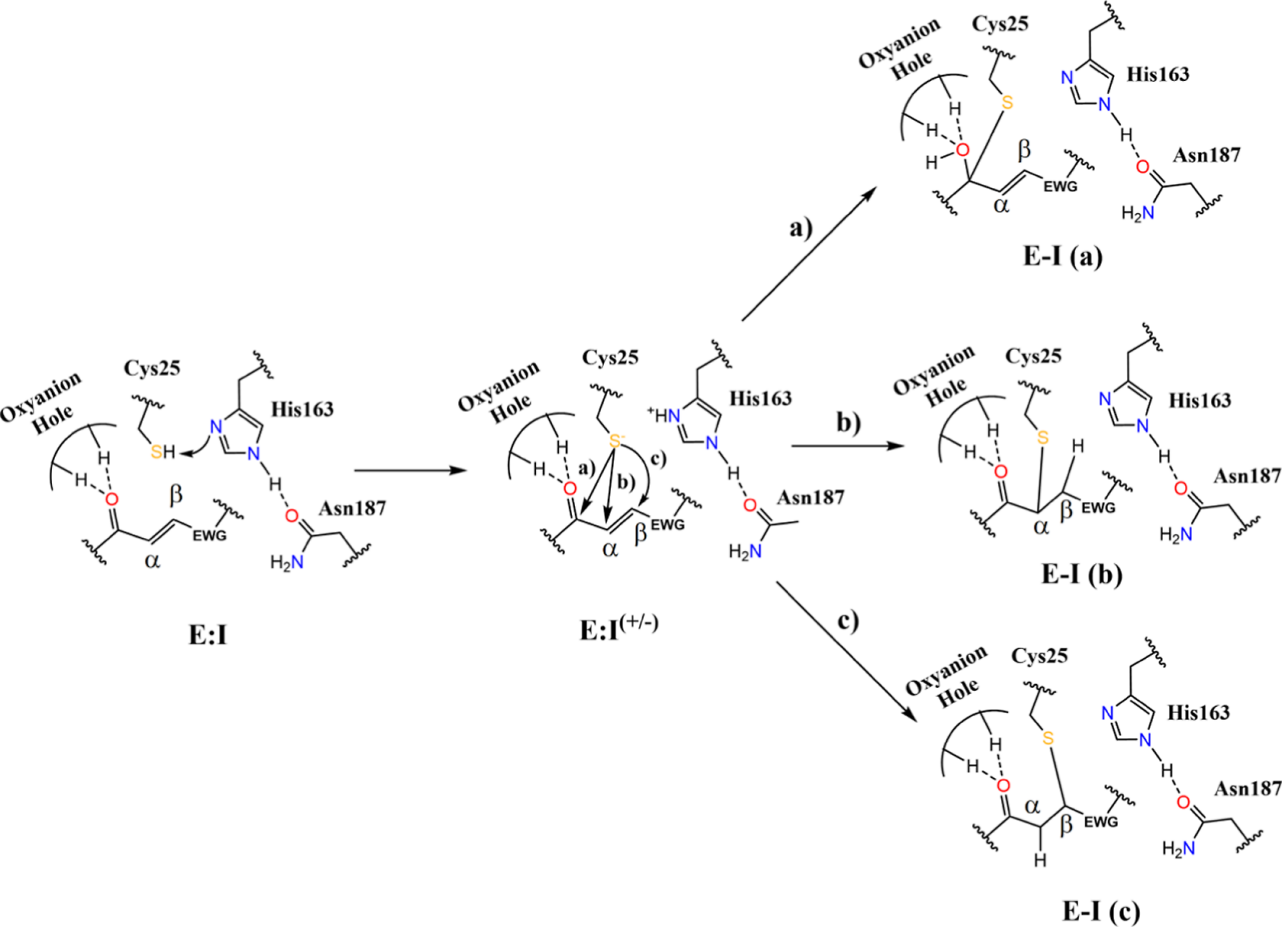
Proposed molecular mechanisms of the chemical step of CatL inhibition including the formation of the activated Cy25s/His163 ion pair (E:I to E:I^(±)^) and the enzyme–inhibitor covalent formation (E:I^(±)^ to E–I) through three possible molecular mechanisms: **a**, **b**, and **c** (see the text for details).

Once the intermediate E:I^(±)^ is formed, the nucleophilic attack of the Cys25 to the inhibitor can take place following three possible routes (Figure 9): the attack to the carbonyl carbon (mechanism “**a**”), the attack to the Cα (mechanism “**b**”), or the attack to the Cβ of the warhead double bond of the inhibitor (mechanism “**c**”). The proton transfer from His163 back to the negatively charged inhibitor was explored in all three possible mechanisms, giving three a priori plausible final E–I complexes. The PESs of all three possible mechanisms were studied independently using M06-2X/MM hybrid potentials (see Figure S10 and the computational details in the Supporting Information for details).

**Figure 10:**
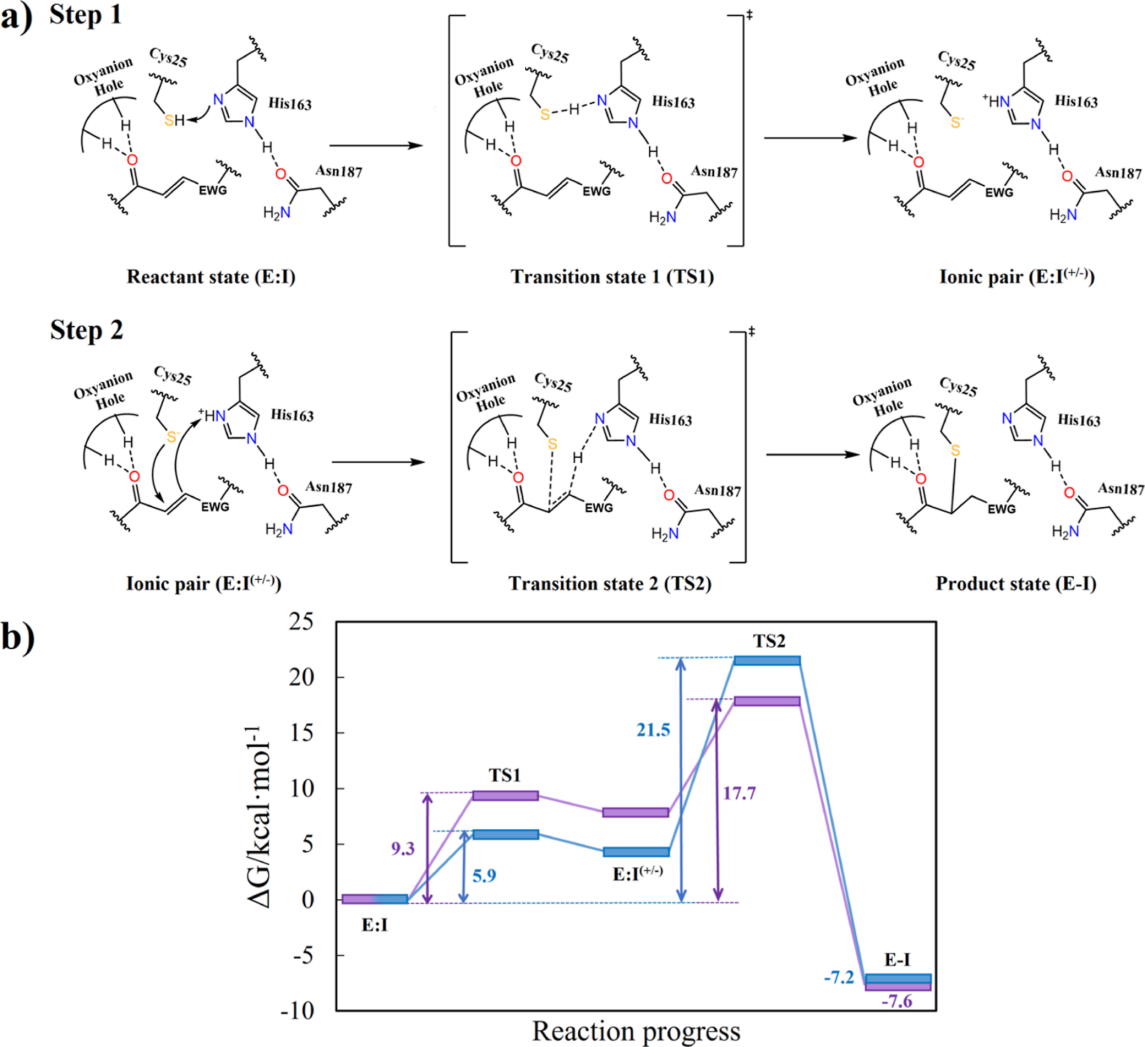
(a) Scheme of the chemical steps of the inhibition mechanism of CatL with **1** and **2** inhibitors. (b) M06-2X/MM free-energy profiles were computed for the CatL inactivation with the **1** (purple) and **2** (blue) inhibitors.

**Figure 11:**
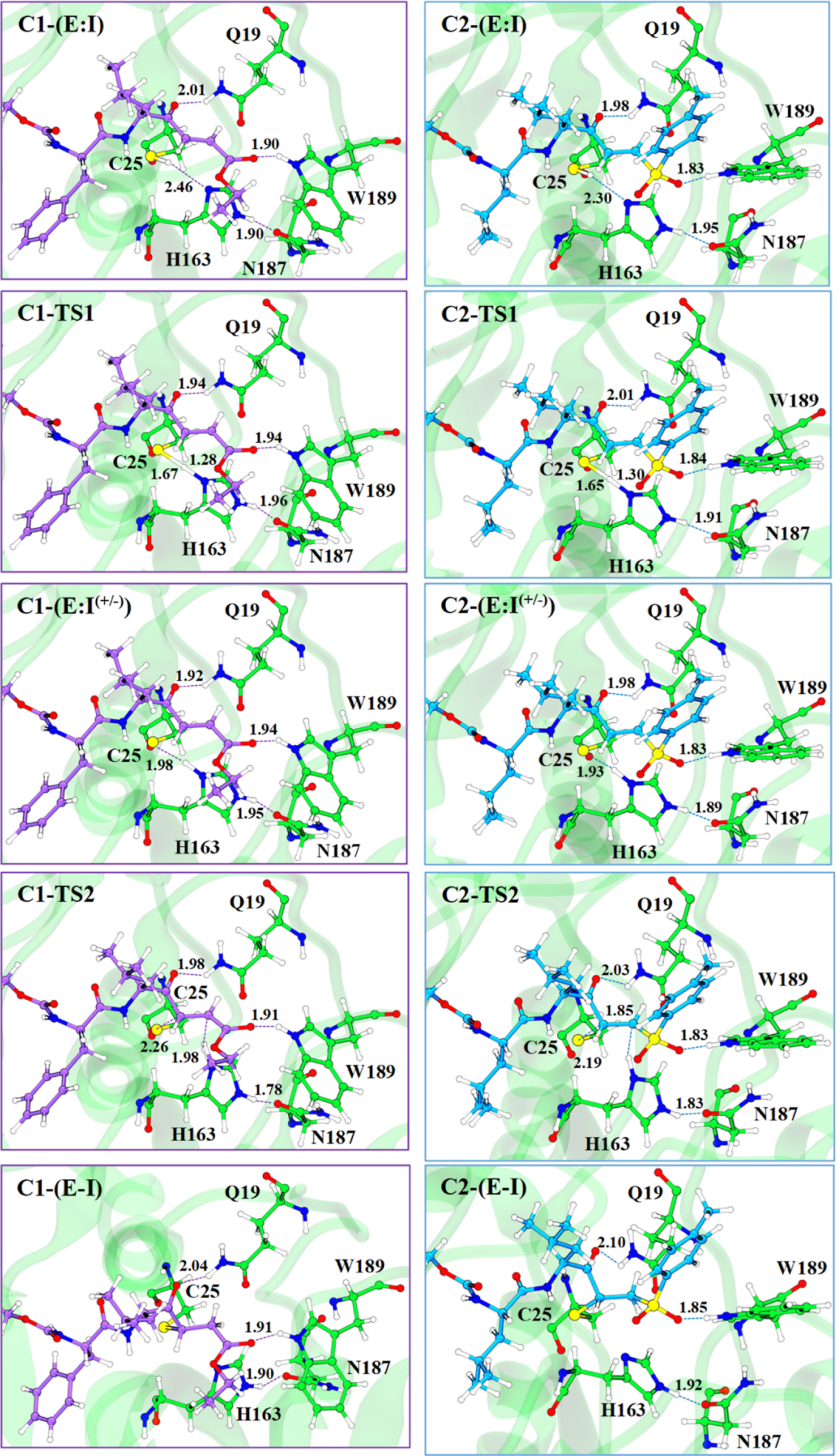
Structures of the stationary point appearing along the inhibition of CatL with **1** (left panels) and **2** (right panels) optimized at the M06-2X/MM level. Key residues and substrates are shown in a ball-and-stick representation. All distances are in Å.

According to the geometrical analysis of the active site and the obtained PESs (see Figure S10), only mechanism “**b**” was feasible. The generated PES allowed one to localize and refine a TS structure at the M06-2X/MM level, connecting the E:I^(±)^ with a product state-like structure (E–I) following mechanism “**b**” (see Figure S10a,b). The charged species that are generated when the sulfur attacks either of the carbon atoms of the warhead in mechanism “**a**” or “**c**” are not stable (see Figure S10c–f). Analysis of the geometries reveals that when the Cys25 attacks C^LEU3^ or Cβ^WAR4^ (mechanism “**a**” or “**c**”), the conformation of the complex does not allow the proton to be transferred from His163 to the carbonyl oxygen or the Cα of the substrate in a stepwise or concerted manner (see Figure S11). Therefore, the free energy landscape was computed just for the reaction of inhibition of CatL with compounds **1** and **2** according to mechanism **b** (Figure S12). The resulting FESs, together with the scheme of the reaction, are shown in Figure 10. Representative structures of the optimized stationary points appearing along the reaction route are shown in Figure 11.

According to the results (Figures 10, 11 and Tables S3–S6), both inhibitors follow the same molecular mechanism. Thus, the sulfur atom is at 2.26 and 2.19 Å of the Cα atom in **TS2** for **1** and **2**, respectively, but the proton is still bound to His163 and far from the acceptor carbon atoms (1.98 and 1.85 Å for **1** and **2**, respectively). From the geometrical analysis of **TS2**, it is observed that the Cα–Cβ distance is in between a double and a single bond, indicating that the product is being formed. Moreover, the analysis of charges indicates that the negative charge in the acceptor carbon atom is higher in **2** than in **1** (−0.96 and −0.65 au in **2** and **1**, respectively), which is in agreement with the fact that a sulfone group delocalizes worse a carbanion than the ester group. Another relevant fact is that His163 must rotate toward the double bond to transfer the proton, losing the interaction with Cys25 but enhancing the interaction with Asn187, according to the distance between Hε2^H163^–Oδ1^N187^.

From the energetics point of view, according to the resulting FESs (Figure 10), the rate-determining step of both reactions corresponds to the second step. The activation free energies are 17.7 and 21.5 kcal mol^–1^ for inhibition with **1** and **2**, respectively. These values are in agreement with the trend of the activation free energies that can be derived from the experimentally measured rate constants listed in Table 1 which, in the framework of the TS theory at 25 °C, are 19.3 and 20.4 kcal mol^–1^, respectively. Our results also match with the experimentally confirmed irreversible character of the mechanism since the barriers for the reverse process are 25.0 and 28.4 kcal mol^–1^ for the inhibition with **1** and **2**, respectively.

Finally, a comparison of the E–I structures with recently solved X-ray high resolution structures of cathepsin L structures (i.e., 8C77, 8OZA, 7QKB, and 7W34) shows no significant differences. In fact, many similarities were found, such as the same H-bond contacts of the peptide-like part of all inhibitors as well as the presence of a H-bond acceptor (a carbonyl or the oxygen of an alcohol group) interacting with the oxyanion hole, especially with Gln19. This agreement provides additional support to our computational predictions.

## 4 Conclusions

In the present work, we studied two inhibitors of CatL, compounds **1** and **2**, that were synthesized, and their inhibitory activities were assayed. Both compounds have a similar structure on the recognition part and mainly differ in the warhead at the carboxyl end: compound **1** contains a KVE and inhibitor **2** a KVS. Both compounds were prepared via a straightforward synthetic procedure from N-protected leucinal. The enzymatic fluorescence assays showed both **1** and **2** to be potent inhibitors of CatL. According to the *in vitro* assays, both compounds inhibit CatL in an irreversible mode. The kinetic constants were measured for both compounds, displaying high *k*_inact_/*K*_*i*_ ratios. The equilibrium constant *K*_*i*_ corresponding to the inverse of the first step of the inhibition process, the formation of the reversible noncovalent E:I complex, was similar in both cases (nanomolar range), but the kinetic constant *k*_inact_ for the second irreversible step was 1 order of magnitude higher for the ester than for the sulfone.

The molecular mechanism of the inhibition process with the two compounds was studied by means of MD simulations with the MM and M06-2X/MM potentials. According to our results, both compounds form a stable reversible E:I complex with a similar pattern of interactions between the inhibitors and the protein active site despite the significant structural and electronic differences between the compounds. The chemical step of the process took place in two steps. The first one consists of the activation of the catalytic Cys25/His163 dyad to form a metastable ion pair formed via a proton transfer from Cys25 to His163. The second step is an asynchronous concerted process corresponding to the nucleophilic attack of the negatively charged Cys25 to the Cα of the double bond of the warhead and the proton transfer from the positively charged His163 to the charged Cβ of the double bond. According to the FESs obtained with the FEP method at the DFT/MM level, the reaction is an exergonic process in both cases. The rate-limiting step corresponds to the second step with overall activation free energies of 17.7 and 21.5 kcal mol^–1^ for the inhibition with **1** and **2**, respectively. These values are in very good agreement with the activation free energies derived from the experimentally measured rate constants of 19.3 and 20.4 kcal mol^–1^, respectively. Our results also match with the experimentally confirmed irreversible character of the mechanism since the barriers for the reverse process are 25.0 and 28.4 kcal mol^–1^ for the inhibition with **1** and **2**, respectively.

Overall, the two designed compounds show promising *in vitro* inhibition activities, which are reflected in the results derived from computer simulations. However, although both theory and *in vitro* assays suggest compound **1** to be slightly more reactive than compound **2**, keeping in mind that the ester group that presents in the warhead of the former is potentially hydrolyzable, lowering its stability in cell media, compound **2** with a sulfone group represents an alternative for future possible developments. The detailed description of the interactions established between the inhibitor and the active site of the protein derived from the computer simulations can be used as a guide for the redesign of more potent and selective inhibitors to be used as drugs with medical applications for the treatment of diverse human pathologies. These could imply introducing a group in P1′ and P2′ to favor the interactions with the corresponding S1′ and S2′ cavities.

## Supporting Information Available

Additional computational details; rmsd computed along the MM MD simulation for the backbone atoms of the protein; FF parameters for inhibitors, rmsd computed along the MM MD simulation for the backbone atoms of the protein, details of the active site and QM-MM partitioning, M06-2X/6-31+G(d,p):AM1/MM and M06-2X/6-31+G(d,p)/MM PESs, description of ^1^H NMR, ^13^C NMR, IR, and HRMS spectra, and ^1^H NMR and ^13^C NMR spectra for all compounds (PDF)
