# Supplementary material for: Impact of the Warhead of Dipeptidyl Keto Michael Acceptors on the Inhibition Mechanism of Cysteine Protease Cathepsin L
Source: ACS Catal. 2023 Oct 3;13(20):13354–68. doi: 10.1021/acscatal.3c02748 (PMC10594577; doi:10.1021/acscatal.3c02748)
Supplement: Supplementary file 1 — cs3c02748_si_001.pdf [file cs3c02748_si_001.pdf]

SUPPLEMENTARY INFORMATION

for

Impact of the Warhead of Dipeptidyl Keto Michael Acceptors on the Inhibition Mechanism of Cysteine Protease Cathepsin L

Adrián Fernández-de-la-Pradilla, Santiago Royo, Tanja Schirmeister, Fabian Barthels, Katarzyna Świderek, Florenci V. González,* Vicent Moliner**

Corresponding Authors:

* F. V. G., e-mail address: fgonzale@uji.es; tel, +34964729156.

* V.M., e-mail address: moliner@uji.es; tel, +34964728084.

* K. Ś., e-mail address: swiderek@uji.es; tel, +34964728070.

Synthesis……………………………………………………………………S2

NMR Spectra…………………………………………………………………S7

Parameters for compounds 1 and 2……………………………………………S11

Results of MM MD simulations………………………………………………S17

Results of QM/MM MD simulations…………………………………………S22

References……………………………………………………………………S28

Synthesis

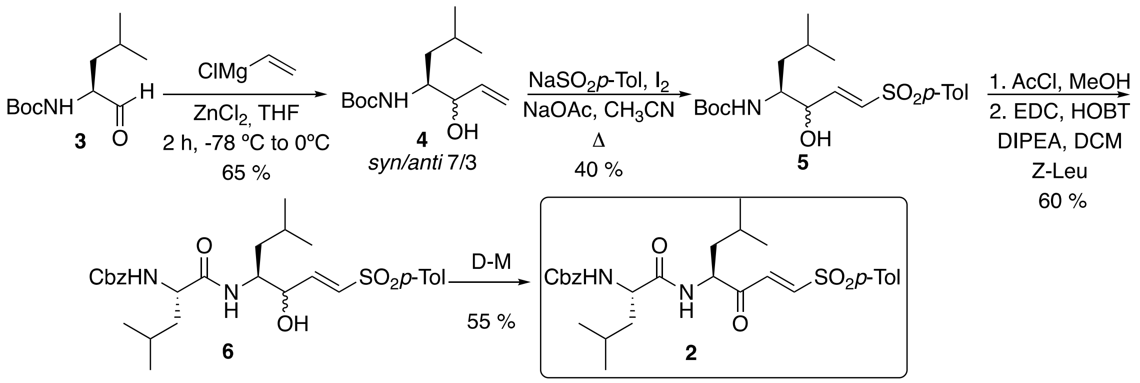

***tert*-Butyl (S)-(1-(methoxy(methyl)amino)-4-methyl-1-oxopentan-2-yl)carbamate.** Boc-L-Leucine monohydrate, 99% (2.132 g, 8.47 mmol), 2-chloro-1-methyl-pyridinium, 97% (2.164 g, 8.47 mmol) and N,O-dimethylhydroxylamine hydrochloride, 98% (0.927 g, 9.31 mmol, 1.1 eq) were disolved in destilled CH_2Cl_2 (94.7 mL, 10 mL/mmol). Et_3N (4.72 mL, 33.88 mmol, 4 eq) was added. The reaction was carried out heating under reflux until completion (overnight). NH_4Cl aqueous saturated solution (30 mL) was added. The phases were separated and the aqueous phase was extracted with CH_2Cl_2 (3 x 20 mL). The organic phases were collected and washed with aqueous saturated solution of $NaHCO_3$ (15 mL). They were dried over $MgSO_4$ and concentrated under vacuum. The reaction crude was purified by liquid chromatography (silica gel, hexane:ethyl acetate, 8:2) to afford a pale yellowish oil (1.813 g, Yield= 78 %).

^{1}H NMR (300 MHz, $CDCl_3$) δ 5.07 (d, *J* = 9.2 Hz, 1H), 4.60 (m, 1H), 3.67 (s, 3H), 3.08 (s, 3H), 1.68 – 1.53 (m, 1H), 1.34 (m, 2H), 1.31 (s, 9H), 0.83 (dd, *J* = 6.7, 6.5 Hz, 6H) ppm.

^{13}C NMR (75 MHz, $CDCl_3$) δ 173.56, 155.39, 78.87, 61.21, 48.68, 41.63, 28.07, 24.42, 23.05, 21.29 ppm.

HRMS (ESI) *m/z* calculated for $C_{13}H_{26}N_2O_4$ $[M+H]^+$: 275.1971, found: 275.1971.

IR (KBr) ν 3296, 2990, 2931, 2915, 2862, 1701, 1652, 1492, 1438, 1370, 1363, 1243, 1163 cm^{-1}.

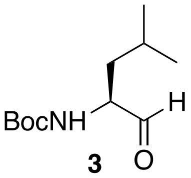

3

***tert*-Butyl (S)-(4-methyl-1-oxopentan-2-yl)carbamate 3.** To a stirred solution of N-methoxy-N-methylamide **2** (1.292 g, 4.71 mmol, 1 eq) in THF (30.6 mL, 6.5 mL/mmol) placed in an ice-bath, $LiAlH_4$ (0.536 g, 14.13 mmol, 3 eq) was slowly added. The reaction was followed by TLC until completion (4 h). Rochelle salt saturated solution was added. The organic phases were extracted with AcOEt (3 x 20 mL), dried over $MgSO_4$ and concentrated under vacuum. The reaction crude was purified by liquid chromatography (silica gel, hexane:ethyl acetate, 9:1) to afford as a clear oil (0.239 g, Yield= 73 %).

^1H NMR (300 MHz, $CDCl_3$) δ 9.58 (s, 1H), 4.92 (s, 1H), 4.23 (m, 1H), 1.79 – 1.72 (m, 1H), 1.45 (s, 9H), 1.43 – 1.32 (m, 2H), 0.98 – 0.95 (m, 6H) ppm.

^{13}C NMR (75 MHz, $CDCl_3$) δ 200.51, 155.75, 80.24, 58.56, 38.30, 28.42, 24.80, 23.22, 22.08 ppm.

HRMS (ESI) *m/z* calculated for $C_{11}H_{21}NO_3$ $[M+Na]^+$: 238.1419, found: 238.1461.

IR (KBr) ν 3310, 2992, 2950, 2921, 2865, 1730, 1682, 1498, 1448, 1373, 1364, 1243, 1160 cm^{-1}.

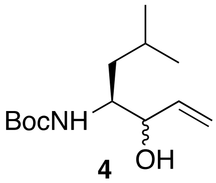

4

syn/anti 7/3

***tert*-Butyl ((4S)-3-hydroxy-6-methylhept-1-en-4-yl)carbamate 4.**

To a stirred solution of aldehyde **3** (0.785 g, 3.65 mmol) in THF (18.25 mL, 5 mL/mmol), $ZnCl_2$ (0.925 g, 7.30 mmol, 2 eq) was added. The reaction was placed in an acetone-liquid N_2 bath (-78 °C) and the vinylmagnesium chloride 1.7M in THF (10.72 mL, 18.23 mmol, 5 eq) was added under N_2 atmosphere. The mixture was stirred for 2 days and 22 h with warming to 0 ºC. HCl 1M (15 mL) was added and the phases were separated. The organic phase was extracted with ethyl acetate (3 x 20 mL) and the extracted washed with brine, dried over $MgSO_4$ and concentrated under vacuum. The reaction crude was purified by liquid chromatography (silica gel, hexane:ethyl acetate, 8:2 to 7:3) to afford a yellowish oil (0.579 g, Yield= 65 %).

R_f (Hex:AcOEt= 8:2)= 0.45.

^{1}H NMR (400 MHz, $CDCl_3$) δ 5.88 (ddd, *J* = 17.2, 10.5, 5.9 Hz, 1H), 5.28 (dt, *J* = 17.2, 1.5 Hz, 1H), 5.18 (dt, *J* = 10.5, 1.4 Hz, 1H), 4.60 (s, 1H), 4.10 – 3.99 (m, 1H), 3.65 (s, *J* = 2.5 Hz, 1H), 2.31 (s, 1H), 1.73 – 1.60 (m, 1H), 1.42 (s, 9H), 1.41 – 1.34 (m, 2H), 0.92 (dd, *J* = 6.6, 1.3 Hz, 6H).

^{13}C NMR (101 MHz, $CDCl_3$) δ 156.54, 138.45, 116.65, 79.43, 75.37, 53.12, 39.31, 28.49, 24.97, 23.44, 22.11 ppm.

HRMS (ESI) *m/z* calculated for $C_{13}H_{25}NO_3$ $[M+H]^+$: 244.1868, found: 244.1913.

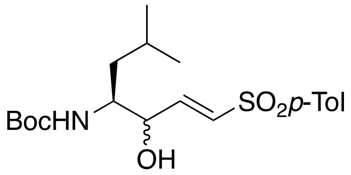

5

***tert*-Butyl ((4S,E)-3-hydroxy-6-methyl-1-tosylhept-1-en-4-yl)carbamate 5.**

Iodine (1.081 g, 4.26 mmol, 3 eq) was added to a suspension mixture of **4** (345 mg, 1.42 mmol), p-toluen sulfinic acid sodium salt, 97%(1.563 g, 8.51 mmol, 6 eq) and NaOAc (349 mg, 4.26 mmol, 3 eq) in CH_3CN (14.2 mL), and the reaction mixture was vigorously stirred at refluxing temperature for 40 hr. The reaction mixture was quenched by the addition of 20 mL of saturated aqueous sodium thiosulfate ($Na_2S_2O_3$) and basified with 20 mL saturated aqueous sodium hydrogen carbonate. The mixture was extracted with ethyl acetate (3 x 20 mL) and the extracted washed with water (20 mL), brine (20 mL), dried over $MgSO_4$ and concentrated under vacuum. The residue was purified by column chromatografhy (silica gel, hexane: ethyl acetate 8:2) to afford a yellow solid (256 mg, 40 %).

^{1}H NMR (400 MHz, $CDCl_3$) δ 7.81 – 7.67 (m, 2H), 7.36 – 7.27 (m, 2H), 6.93 (ddd, *J* = 18.4, 15.5, 8.2 Hz, 1H), 6.69 – 6.58 (m, 1H), 4.96 – 4.59 (m, 1H), 4.41 (dd, *J* = 41.0, 23.6 Hz, 1H), 3.66 (s, 1H), 2.43 – 2.38 (m, 3H), 1.72 – 1.52 (m, 1H), 1.51 – 1.28 (m, 11H), 0.93 – 0.81 (m, 6H).

13C NMR (101 MHz, CDCl3) δ 156.44 (s), 145.90 (s), 144.44 (d, J = 10.8 Hz), 137.42 (s), 131.36 (s), 129.95 (s), 127.77 (s), 79.95 (s), 77.48 (s), 77.16 (s), 76.84 (s), 72.52 (s), 53.04 (s), 39.82 (s), 28.34 (s), 24.88 (s), 23.20 (s), 21.63 (s).

HRMS (ESI) *m/z* calculated for $C_{14}H_{27}NO_3$ $[M+Na]^+$: 420.1821, found: 420.1820.

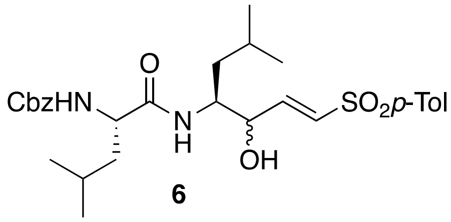

6

Benzyl ((2*S*)-1-(((4*S*,*E*)-3-hydroxy-6-methyl-1-tosylhept-1-en-4-yl)amino)-4-methyl-1-oxopentan-2-yl)carbamate 6. To an ice-bath cold methanol (1 mL) was added acetyl chloride (496 mL, 6.8 mmol). Then a solution of compound **5** (174 mg, 0.45 mmol) in methanol (0.57 mL, 1.3 mL/mmol) was added and the resulting mixture was stirred at room temperature (25 °C) for 30 min. After this time the reaction mixture was concentrated under vaccum and the resulting reaction crude was submitted to the next step without any further purification. The dried crude from previous step was dissolved in dichloromethane (4.5 mL) and the resulting mixture was cold with an ice-bath. Then benzyloxycarbonyl leucine (133 mg, 0.5 mmol), hydroxybenzotriazole (68 mg, 0.5 mmol), triethyl amine (250 mL, 1.8 mmol) and EDC (78 mg, 0.5 mmol) were sequentially added. The resulting mixture was stirred at 23 °C for 8 hours and then was quenched with saturated ammonium chloride aqueous solution (25 mL) and extracted with dichloromethane (3 x 15 mL), the organic layers were washed with 1M HCl solution (15 mL), then with saturated sodium hydrogen carbonate aqueous solution (15 mL) and then with brine (15 mL), dried (Na_2SO_4) and concentrated. The residue was purified by column chromatografhy (silica gel, hexane: ethyl acetate 8:2) to afford a yellow oil (147 mg, 60 %).

^{1}H NMR (400 MHz, $CDCl_3$) δ 7.68 (d, *J* = 8.3 Hz, 2H), 7.23 – 7.29 (m, 9H), 6.83 (dd, *J* = 14.9, 3.6 Hz, 1H), 6.55 (dd, *J* = 14.9, 1.4 Hz, 1H), 6.33 (d, *J* = 8.2 Hz, 1H), 5.04 (s, 2H), 4.27 (m, 1H), 3.97 (ddd, *J* = 9.5, 7.9, 4.9 Hz, 1H), 3.78 (ddd, *J* = 12.9, 8.8, 4.5 Hz, 1H), 2.34 (s, 3H), 1.46 – 1.58 (m, 4H), 1.29 -1.38 (m, 2H), 0.84 (d, *J* = 6.4 Hz, 3H), 0.82 (d, *J* = 6.2 Hz, 3H), 0.79 (d, *J* = 6.3 Hz, 3H) ppm.

13C NMR (101 MHz, CDCl3) δ 172.3, 156.4, 145.5, 144.4, 137.4, 136.0, 131.6, 129.9, 128.6, 128.2, 127.9, 72.5, 67.4, 53.7, 52.8, 40.9, 39.4, 24.8, 23.0, 22.9, 21.8, 21.6 ppm.

HRMS (ESI) calculated for M + H^+ (C29H41N2O6S): 545.2680, found: 545.2686.

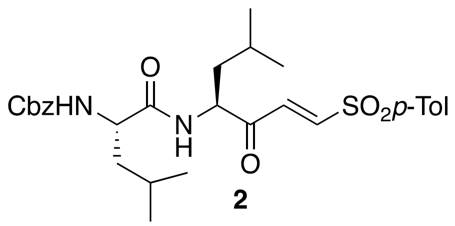

2

Benzyl ((*S*)-4-methyl-1-(((*S*,*E*)-6-methyl-3-oxo-1-tosylhept-1-en-4-yl)amino)-1-oxopentan-2-yl)carbamate 2. To an ice-bath cold solution of compound **6** (98 mg, 0.18 mmol) in dichloromethane (10 mL)) was added Dess-Martin periodinane (mg, mmol). The resulting mixture was stirred at room tempearture for 3.5 h. Then a saturated aqueous solution of $Na_2S_2O_3$/$NaHCO_3$ (10 mL) was added and stirred for 15 min. Then the mixture was extracted with dichloromethane (3 x 15 mL), the organic layers were washed with brine (15 mL), dried (Na_2SO_4) and concentrated. The residue was purified by column chromatografhy (silica gel, hexane: ethyl acetate 8:2) to afford a yellow oil (58 mg, 60 %).

^{1}H NMR (400 MHz, $CDCl_3$) δ 7.71 (d, *J* = 8.3 Hz, 1H), 7.30 (d, *J* = 8.0 Hz, 1H), 7.19 (m, 5H), 7.20 (d, *J* = 15.1 Hz, 1H), 7.08 (d, *J* = 15.1 Hz, 1H), 6.48 (d, *J* = 5.7 Hz, 1H), 5.08 (d, *J* = 8.2 Hz, 1H), 5.04 (s, 2H), 4.66 (ddd, *J* = 9.6, 7.6, 3.9 Hz, 1H), 4.11 (m, 1H), 2.38 (s, 3H), 1.30- 1.60 (m, 6H), 0.82 – 0.87 (m, 12H) ppm.

13C NMR (101 MHz, CDCl3) δ 196.1, 187.0, 172.3, 156.3, 145.7, 142.3, 136.1, 135.4, 132.2, 130.3, 128.6, 128.4, 128.3, 128.0, 67.2, 56.6, 53.4, 41.1, 39.5, 29.7, 24.9, 24.7, 23.1, 22.8, 22.0, 21.7, 21.6 ppm.

HRMS (ESI) calculated for M + H^+ (C29H39N2O6S): 543.2524, found: 543.2529.

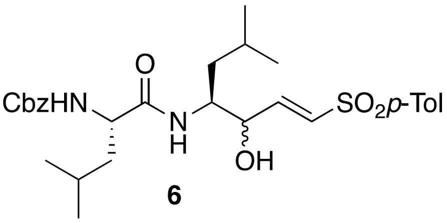
CbzHN
O
N
H
OH
SO2p-Tol

6

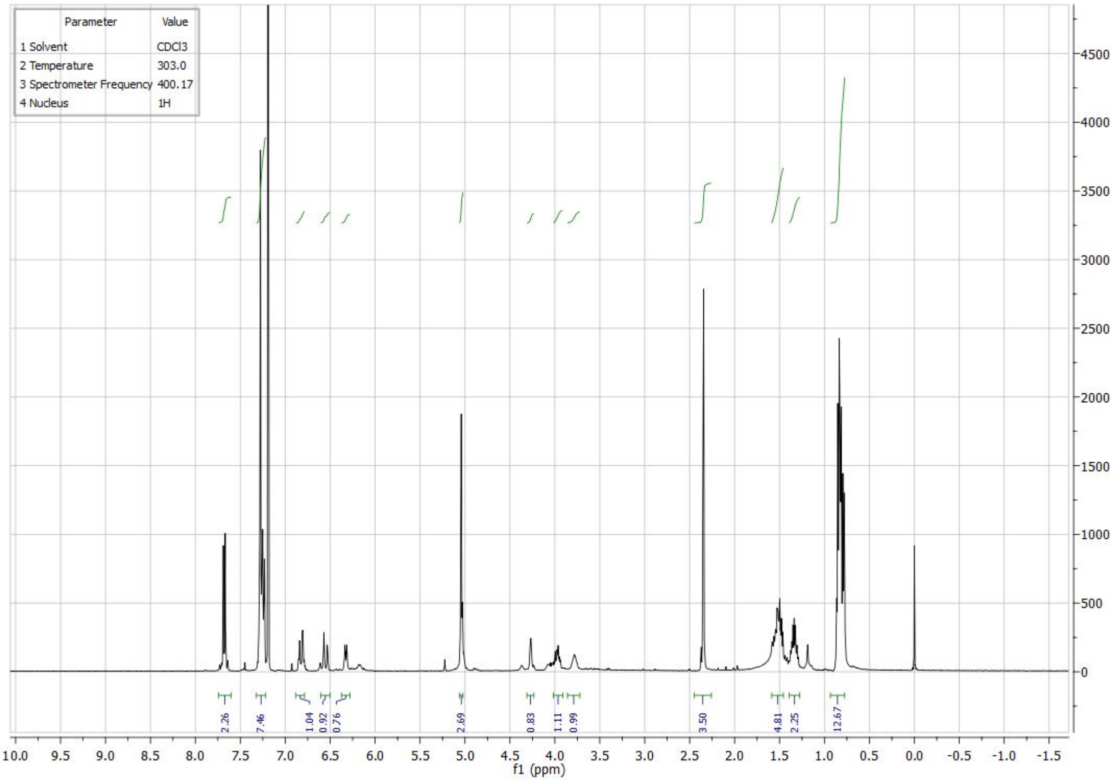
Parameter Value
1 Solvent CDCl3
2 Temperature 303.0
3 Spectrometer Frequency 400.17
4 Nucleus 1H
2.26
7.46
1.04
0.92
0.76
2.69
0.83
1.11
0.99
3.50
4.81
2.25
12.67
f1 (ppm)

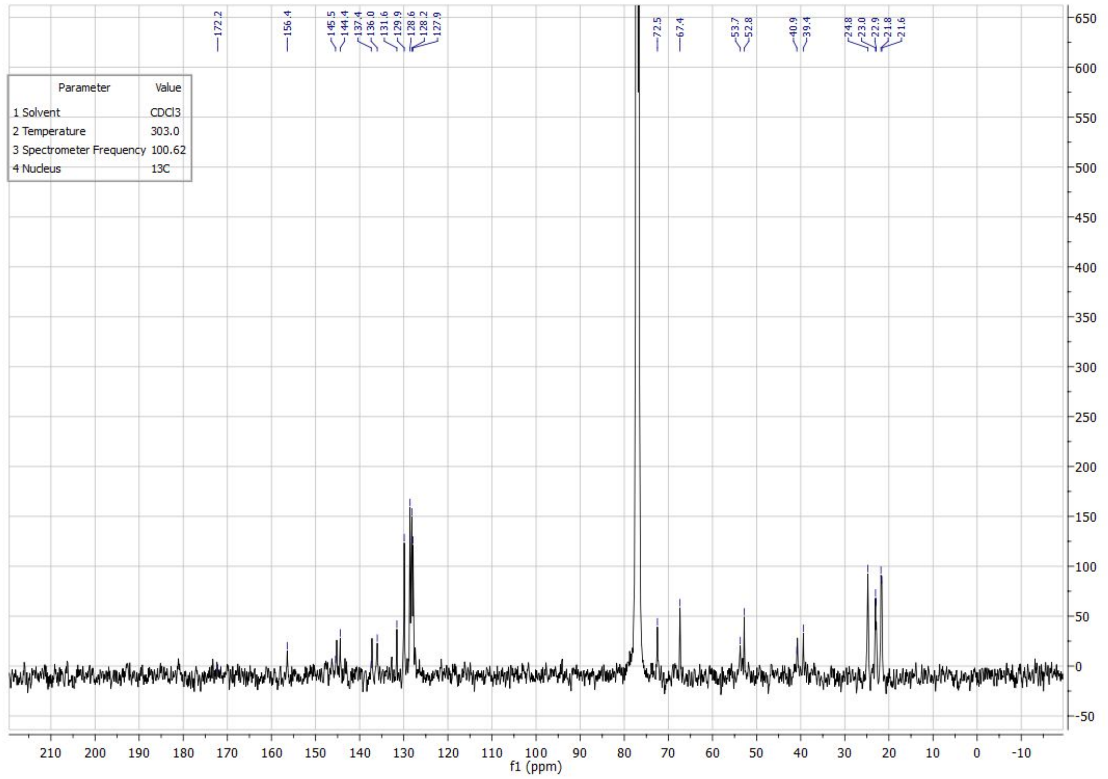
172.2
156.4
145.5
144.4
137.4
136.0
131.6
129.9
128.6
128.2
127.9
72.5
67.4
53.7
52.8
40.9
39.4
24.8
23.0
22.9
21.8
21.6
Parameter Value
1 Solvent CDCl3
2 Temperature 303.0
3 Spectrometer Frequency 100.62
4 Nucleus 13C
f1 (ppm)

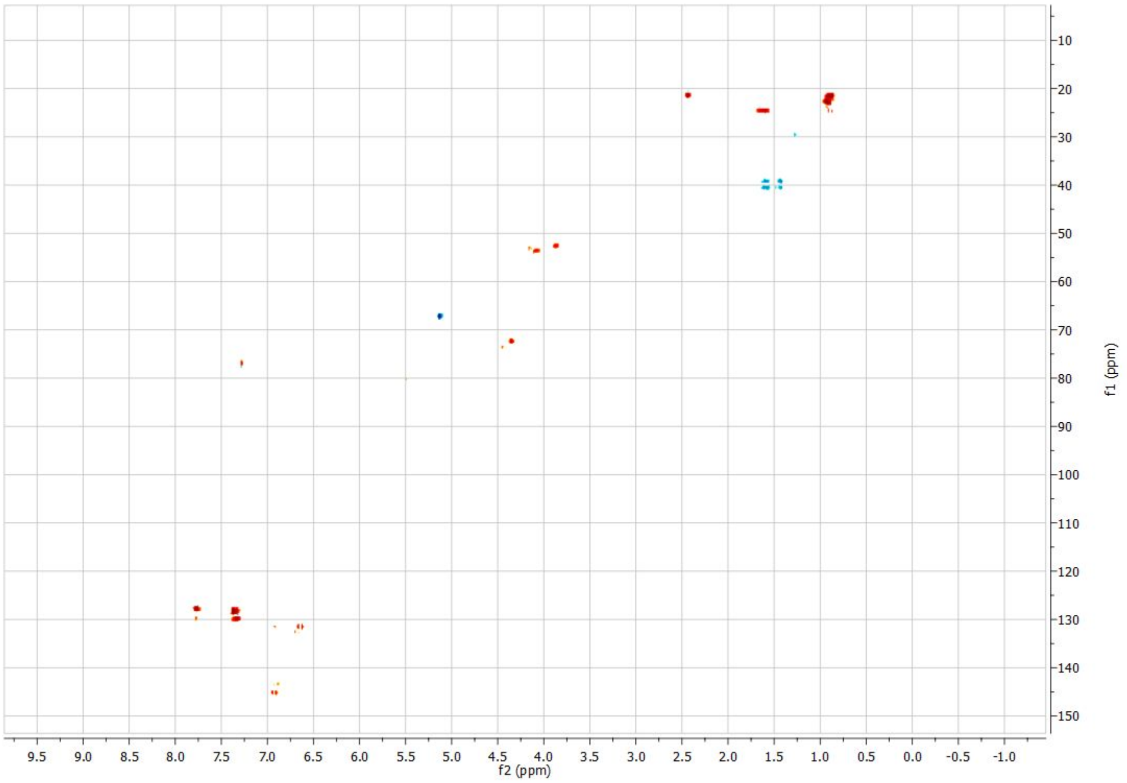
f1 (ppm)
f2 (ppm)

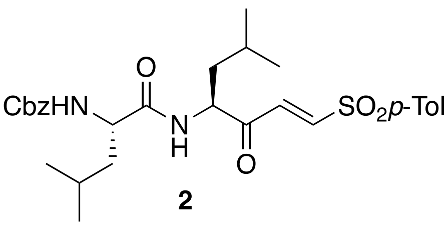
CbzHN
N
H
O
O
SO2p-Tol

2

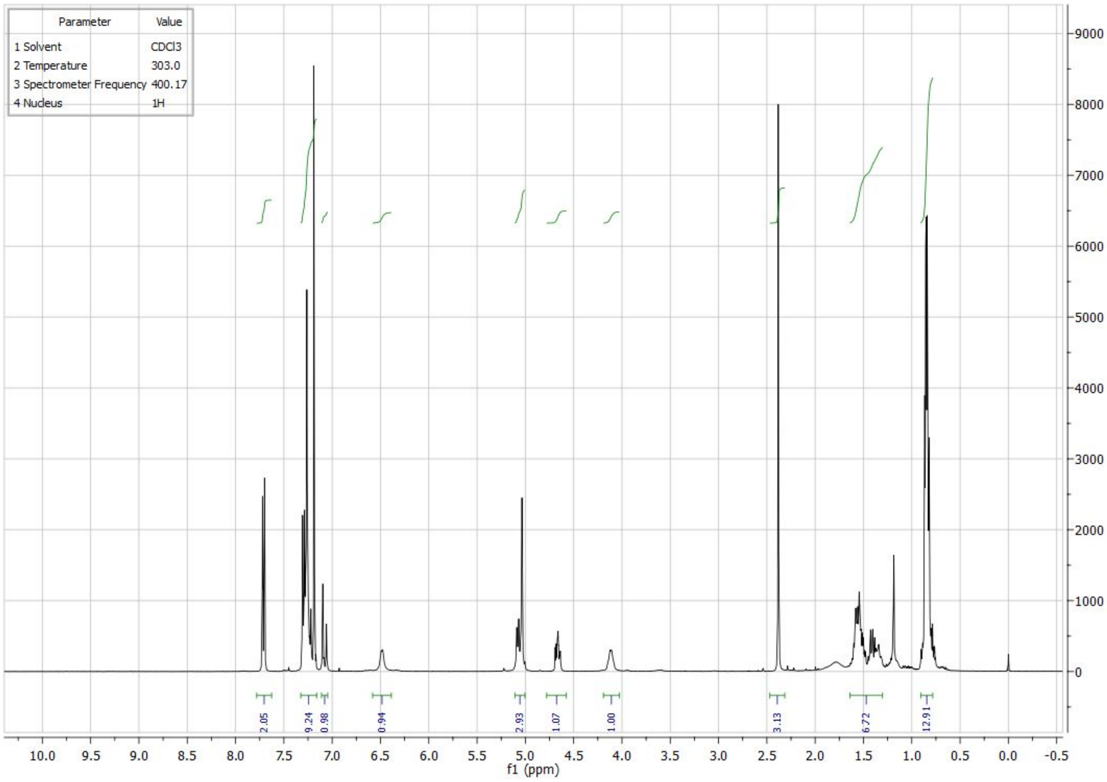
Parameter Value
1 Solvent CDCl3
2 Temperature 303.0
3 Spectrometer Frequency 400.17
4 Nucleus 1H
2.05
9.24
0.98
0.94
2.93
1.07
1.00
3.13
6.72
12.91
f1 (ppm)

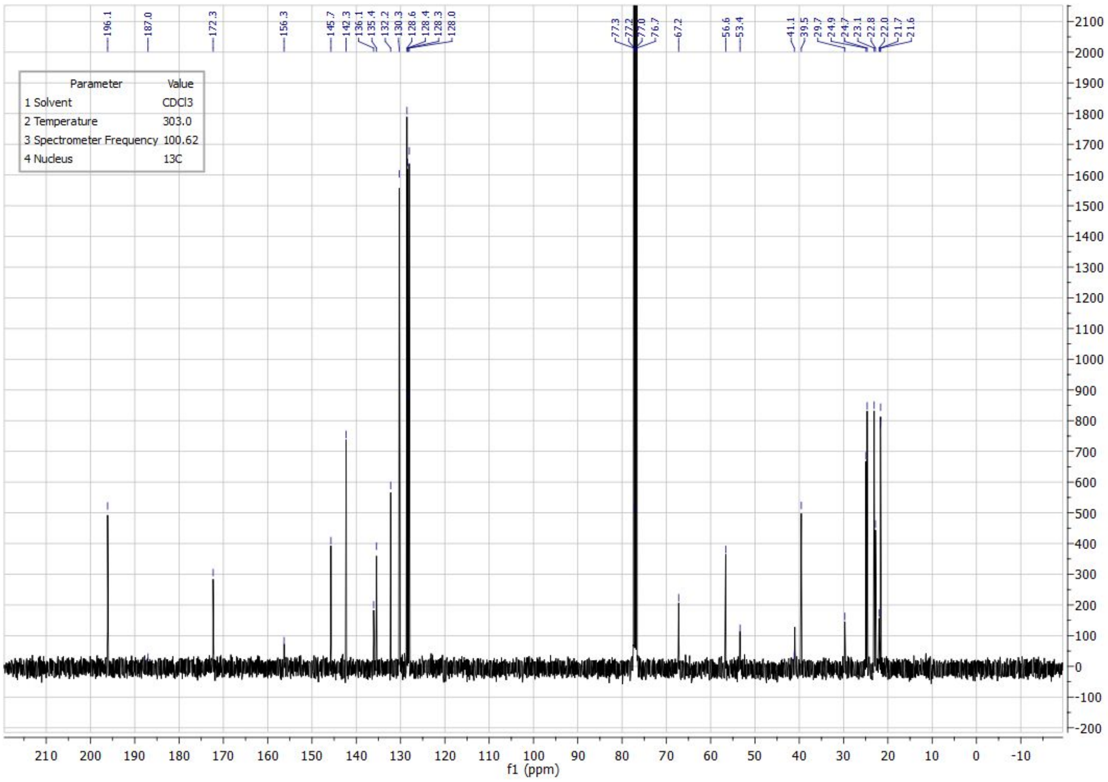
196.1
187.0
172.3
156.3
145.7
142.3
136.1
135.4
132.2
130.3
128.6
128.4
128.3
128.0
77.3
77.2
77.0
76.7
67.2
56.6
53.4
41.1
39.5
29.7
24.9
24.7
23.1
22.8
22.0
21.7
21.6
Parameter Value
1 Solvent CDCl3
2 Temperature 303.0
3 Spectrometer Frequency 100.62
4 Nucleus 13C
f1 (ppm)

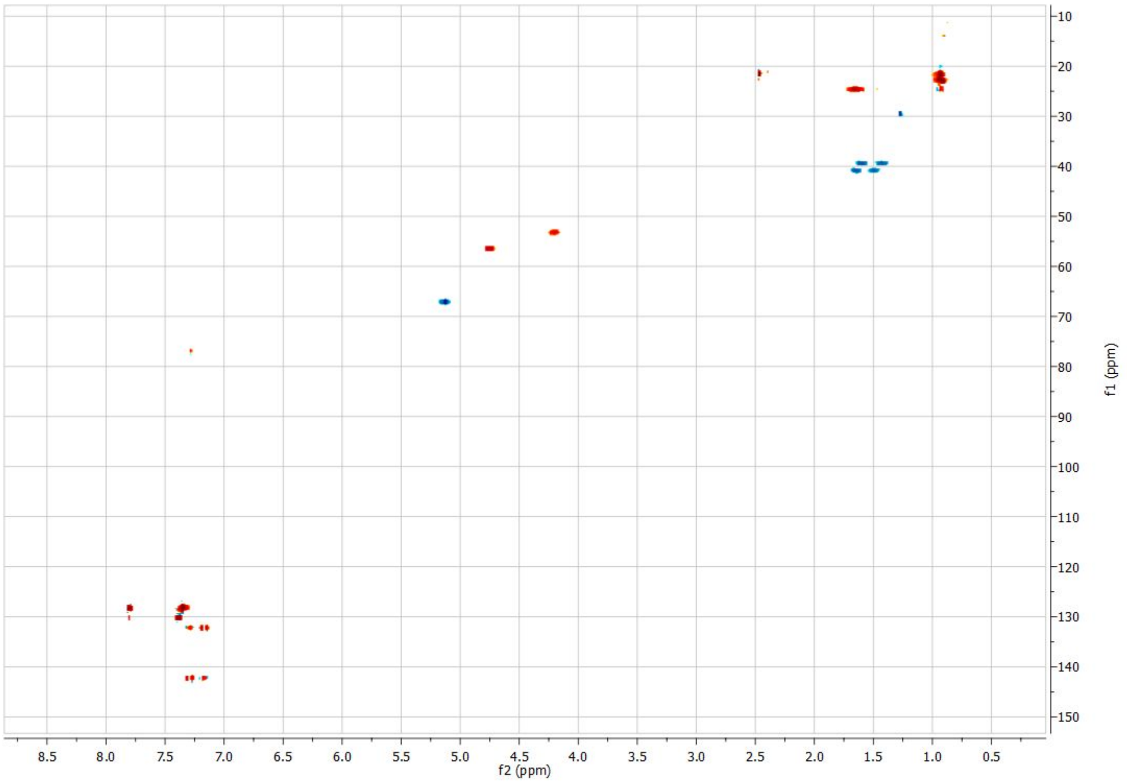

f2 (ppm)
f1 (ppm)

Parameters for compounds 1 and 2. Compound **1** (top) and **2** (bottom) were divided in four parts (see **Figure 4** of main text). The WAR, WAS and CBZ parts were parametrized with Gaff forcefield, and the atoms were named as it is shown. The rest was parametrized with AMBER general force field for amino acids.

Table S1. Atom types, charges and parameters for bonding and non-bonding interactions obtained for WAR, CBZ and WAS of both inhibitors computed using Antechamber software.

Warhead KVE					
**Atom name**	**Atom type**	**Charge**	**Atom name**	**Atom type**	**Charge**
Cα	c2	-0.125803	Cζ	c3	-0.12018
H1	ha	0.136626	H5	hc	0.051684
Cβ	ce	-0.201285	H6	hc	0.051684
Cγ	c	0.658842	H7	hc	0.051684
Oγ	o	-0.521322	H3	h1	0.064596
Oδ	os	-0.418296	H4	h1	0.064596
Cε	c3	0.145944	H2	ha	0.161229
Parameters:					
**Mass**			**Bond**		
c2	12.01	0.36	c2-ha	343.1	1.088
ha	1.008	0.135	c2-ce	547.3	1.346
ce	12.01	0.36	c-ce	354.5	1.482
c	12.01	0.616	ce-ha	342.5	1.088
o	16	0.434	c-o	637.7	1.218
os	16	0.465	c-os	390.8	1.358
c3	12.01	0.878	c3-os	308.6	1.432
hc	1.008	0.135	c3-c3	300.9	1.538
h1	1.008	0.135	c3-h1	330.6	1.097
			c3-hc	330.6	1.097
			C-c2	354.5	1.482
**Angle**			**Angle**		
c-ce-c2	65.5	120.42	h1-c3-os	50.8	109.78
c2-ce-ha	49.6	119.94	c3-c3-hc	46.3	109.8
ce-c2-ha	49.5	120.45	c3-c3-h1	46.4	109.56
ce-c-o	68.8	123.2	hc-c3-hc	39.4	107.58
ce-c-os	70	110.93	h1-c3-h1	39.2	108.46
c-ce-ha	46.5	116.46	c2-C-O	68.8	123.2
c-os-c3	63.3	115.98	C-c2-ha	46.5	116.46
o-c-os	75.3	123.25	C-c2-ce	65.5	120.42
c3-c3-os	68	107.97	CT-C-c2	62.9	116.44
**Dihedrals**					
o-c-ce-c2		4	8.7	180	2

os-c-ce-c2	4	8.7	180	2
ha-c2-ce-c	4	26.6	180	2
ha-c2-ce-ha	4	26.6	180	2
ce-c-os-c3	2	5.4	180	2
c3-c3-os-c	1	0.383	0	-3
c3-c3-os-c	1	0.8	180	1
h1-c3-os-c	3	1.15	0	3
o-c-os-c3	1	2.7	180	-2
o-c-os-c3	1	1.4	180	1
hc-c3-c3-os	1	0	0	-3
hc-c3-c3-os	1	0.25	0	1
h1-c3-c3-hc	9	1.4	0	3
o-c-ce-ha	4	8.7	180	2
os-c-ce-ha	4	8.7	180	2
O-C-c2-ha	4	8.7	180	2
O-C-c2-ce	4	8.7	180	2
CT-C-c2-ha	4	8.7	180	2
CT-C-c2-ce	4	8.7	180	2
**Impropers**				
c-c2-ce-ha	1.1	180	2	
ce-o-c-os	1.1	180	2	

**Nonbonding**			**Nonbonding**		
c2	1.908	0.086	os	1.6837	0.17
ha	1.459	0.015	c3	1.908	0.1094
ce	1.908	0.086	hc	1.487	0.0157
c	1.908	0.086	h1	1.387	0.0157
o	1.6612	0.21			

**Cbz group**					
**Atom name**	**Atom type**	**Charge**	**Atom name**	**Atom type**	**Charge**
Oα	os	-0.432194	H6	ha	0.136215
Cβ	c3	0.184044	H5	ha	0.135191
Cγ	ca	-0.122271	H4	ha	0.136215
Cδ1	ca	-0.105389	H3	ha	0.146457
Cε1	ca	-0.132713	H1	h1	0.072409
Cζ	ca	-0.116123	H2	h1	0.072409
Cε2	ca	-0.132713	C	c	0.649223
Cδ2	ca	-0.105389	O	o	-0.531826
H7	ha	0.146457			
Parameters:					
**Mass**			**Bond**		
os	16	0.465	c3-os	308.6	1.432

c3	12.01	0.878	c-os	390.8	1.358
ca	12.01	0.36	c3-ca	321	1.516
ha	1.008	0.135	c3-h1	330.6	1.097
h1	1.008	0.135	ca-ca	461.1	1.398
c	12.01	0.616	ca-ha	345.8	1.086
o	16	0.434	c-o	637.7	1.218
			c-N	427.6	1.379
**Angle**			**Angle**		
ca-c3-os	68.3	108.95	ca-ca-ha	48.2	119.88
h1-c3-os	50.8	109.78	h1-c3-h1	39.2	108.46
o-c-os	75.3	123.25	N-c-o	74.2	123.05
c-os-c3	63.3	115.98	c-N-H	48.3	117.55
c3-ca-ca	63.5	120.77	c-N-CT	63.4	120.69
ca-c3-h1	47	109.56	N-c-os	74.3	112.82
ca-ca-ca	66.6	120.02			
**Dihedrals**					
os-c3-ca-ca		6	0	0	2
o-c-os-c3		1	2.7	180	-2
o-c-os-c3		1	1.4	180	1
c3-ca-ca-ca		4	14.5	180	2
c3-ca-ca-ha		4	14.5	180	2
ca-ca-ca-ca		4	14.5	180	2
ca-ca-ca-ha		4	14.5	180	2
ha-ca-ca-ha		4	14.5	180	2
h1-c3-ca-ca		6	0	0	2
ca-c3-os-c		3	1.15	0	3
h1-c3-os-c		3	1.15	0	3
o-c-N-H		1	2.5	180	-2
o-c-N-H		1	2	0	1
o-c-N-CT		4	10	180	2
os-c-N-H		4	10	180	2
os-c-N-CT		4	10	180	2
**Impropers**					
c3-ca-ca-ca		1.1	180	2	
ca-ca-ca-ha		1.1	180	2	
**Nonbonding**			**Nonbonding**		
os	1.6837	0.17	h1	1.387	0.0157
c3	1.908	0.1094	c	1.908	0.086
ca	1.908	0.086	o	1.6612	0.21
ha	1.459	0.015			

Warhead KVS					
**Atom name**	**Atom type**	**Charge**	**Atom name**	**Atom type**	**Charge**
Cα	c2	-0.017558	Cδ1	ca	-0.012681
H1	ha	0.129095	H6	ha	0.156758
Cβ	ce	-0.450751	H5	ha	0.144463
Sβ	sy	1.470449	Cω	c3	-0.066135
Oβ2	o	-0.644083	H7	hc	0.054335
Oβ1	o	-0.644083	H8	hc	0.054335
Cγ	ca	-0.374081	H9	hc	0.054335
Cδ2	ca	-0.012681	H4	ha	0.144463
Cε2	ca	-0.15607	H3	ha	0.156758
Cζ	ca	-0.009072	H2	h4	0.178273
Cε1	ca	-0.15607			
Parameters:					
**Mass**			**Bond**		
c2	12.01	0.36	c2-ha	343.1	1.088
ha	1.008	0.135	c2-ce	547.3	1.346
ce	12.01	0.36	ce-sy	245.1	1.788
sy	32.06	2.9	ce-h4	337.8	1.092
o	16	0.434	o-sy	493	1.466
ca	12.01	0.36	ca-sy	243.4	1.791
c3	12.01	0.878	ca-ca	461.1	1.398
hc	1.008	0.135	ca-ha	345.8	1.086
h4	1.008	0.135	c3-ca	321	1.516
			c3-hc	330.6	1.097
			C-c2	354.5	1.482
**Angle**			**Angle**		
c2-ce-sy	61.8	120.2	ca-ca-ca	66.6	120.02
c2-ce-h4	48.6	124.55	ca-ca-ha	48.2	119.88
ce-c2-ha	49.5	120.45	c3-ca-ca	63.5	120.77
ce-sy-o	65.8	108.38	ca-c3-hc	46.8	110.47
ca-sy-ce	60.9	102.78	hc-c3-hc	39.4	107.58
h4-ce-sy	42.2	115	c2-C-O	68.8	123.2
ca-ca-sy	61.5	119.42	C-c2-ha	46.5	116.46
o-sy-o	72.5	121.41	C-c2-ce	65.5	120.42
ca-sy-o	65.8	108.35	CT-C-c2	62.9	116.44
**Dihedrals**					
c2-ce-sy-o		6	7.6	180	2
c2-ce-sy-ca		6	7.6	180	2
ha-c2-ce-sy		4	26.6	180	2
ha-c2-ce-h4		4	26.6	180	2
ca-ca-sy-ce		6	7.8	180	2
ca-ca-ca-sy		4	14.5	180	2

ha-ca-ca-sy		4	14.5	180	2
ca-ca-sy-o		6	7.8	180	2
ca-ca-ca-ca		4	14.5	180	2
ca-ca-ca-ha		4	14.5	180	2
c3-ca-ca-ca		4	14.5	180	2
hc-c3-ca-ca		6	0	0	2
ha-ca-ca-ha		4	14.5	180	2
c3-ca-ca-ha		4	14.5	180	2
h4-ce-sy-o		6	7.6	180	2
h4-ce-sy-ca		6	7.6	180	2
O-C-c2-ha		4	8.7	180	2
O-C-c2-ce		4	8.7	180	2
CT-C-c2-ha		4	8.7	180	2
CT-C-c2-ce		4	8.7	180	2
**Impropers**					
c2-h4-ce-sy		1.1	180	2	
ca-ca-ca-sy		1.1	180	2	
ca-ca-ca-ha		1.1	180	2	
c3-ca-ca-ca		1.1	180	2	
**Nonbonding**			**Nonbonding**		
c2	1.908	0.086	ca	1.908	0.086
ha	1.459	0.015	c3	1.908	0.1094
ce	1.908	0.086	hc	1.487	0.0157
sy	2	0.25	h4	1.409	0.015
o	1.6612	0.21			

Table S2. pKa values of the titratable residues predicted from the empirical program PropKa v.3.0.3.

Residue		pKa	pKmodel	Residue		pKa	pKmodel
ASP	6A	0.92	3.8	HIS	208A	6.29	6.5
ASP	55A	2.34	3.8	TYR	12A	10	10
ASP	71A	2.81	3.8	TYR	72A	10	10
ASP	79A	3.58	3.8	TYR	76A	9.99	10
ASP	84A	3.72	3.8	TYR	89A	13.03	10
ASP	109A	4.76	3.8	TYR	91A	9.41	10
ASP	114A	5.02	3.8	TYR	100A	10.89	10
ASP	137A	1.98	3.8	TYR	104A	9.27	10
ASP	155A	3.8	3.8	TYR	146A	10.02	10
ASP	160A	4.08	3.8	TYR	151A	11.3	10
ASP	162A	3.04	3.8	TYR	170A	10.89	10
ASP	178A	3.87	3.8	TYR	182A	10.39	10
ASP	204A	2.69	3.8	TYR	198A	8.7	10
GLU	9A	4.64	4.5	TYR	217A	10	10
GLU	35A	0.05	4.5	LYS	17A	15.28	10.5
GLU	50A	2.12	4.5	LYS	41A	10.29	10.5
GLU	63A	4.5	4.5	LYS	99A	9.73	10.5
GLU	86A	6.86	4.5	LYS	103A	10.29	10.5
GLU	87A	4.5	4.5	LYS	117A	10.5	10.5
GLU	92A	5.27	4.5	LYS	120A	10.22	10.5
GLU	95A	4.68	4.5	LYS	124A	10.43	10.5
GLU	96A	4.05	4.5	LYS	147A	10.22	10.5
GLU	119A	3.77	4.5	LYS	181A	10.5	10.5
GLU	141A	4.64	4.5	LYS	186A	10.69	10.5
GLU	148A	4.64	4.5	LYS	200A	10.08	10.5
GLU	153A	3.03	4.5	LYS	203A	10.15	10.5
GLU	159A	4.5	4.5	ARG	3A	12.43	12.5
GLU	173A	3.62	4.5	ARG	8A	12.08	12.5
GLU	176A	4.78	4.5	ARG	40A	11.94	12.5
GLU	191A	4.55	4.5	ARG	44A	12.15	12.5
GLU	192A	4.45	4.5	ARG	205A	11.8	12.5
HIS	140A	7.09	6.5	ARG	206A	12.36	12.5
HIS	163A	10.79	6.5				

Results of MM MD simulations

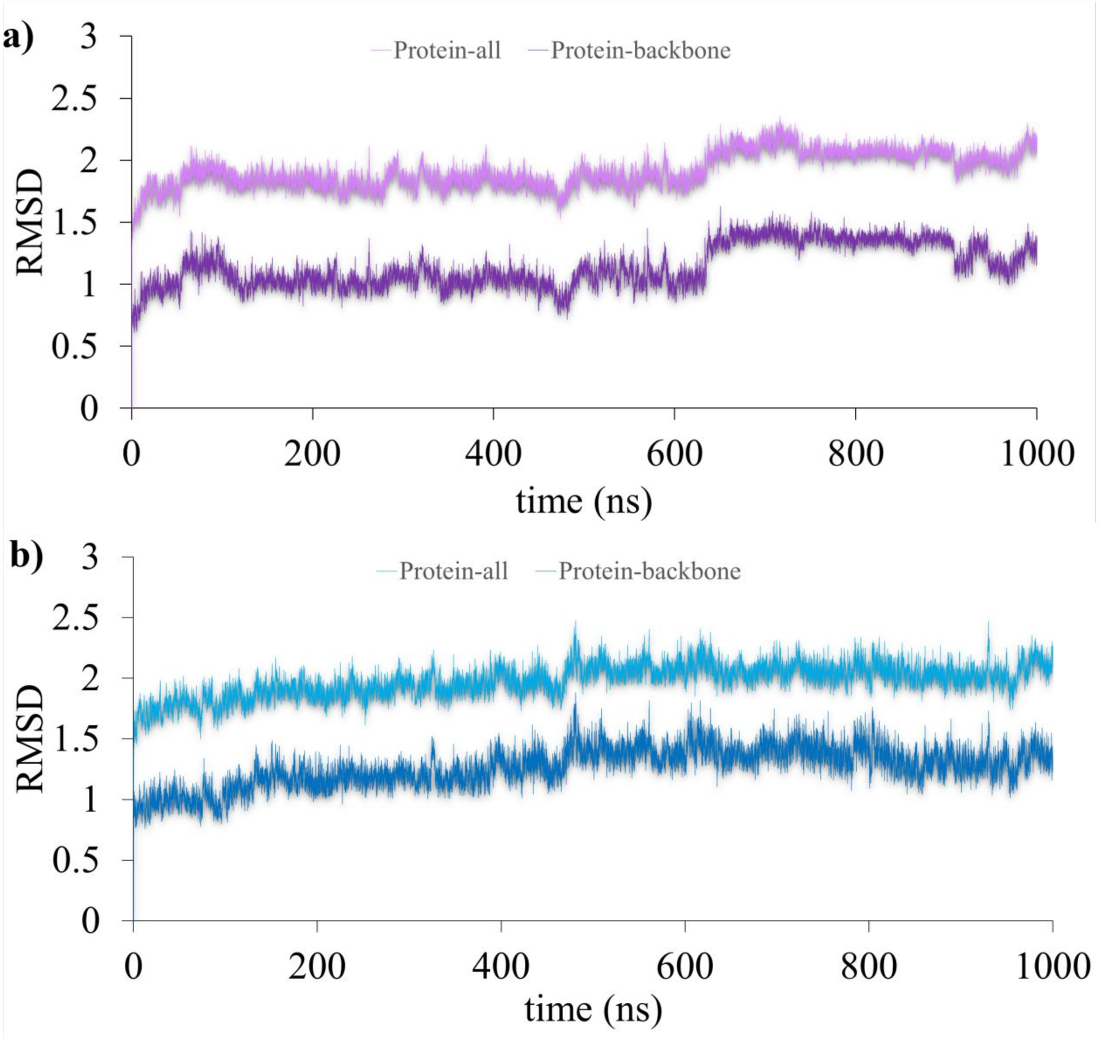

Figure S1. Time evolution of the RMSD (in Å) computed with all the protein atoms and only the protein backbone along the classical MD for **a)** compound **1**, and **b)** compound **2**.

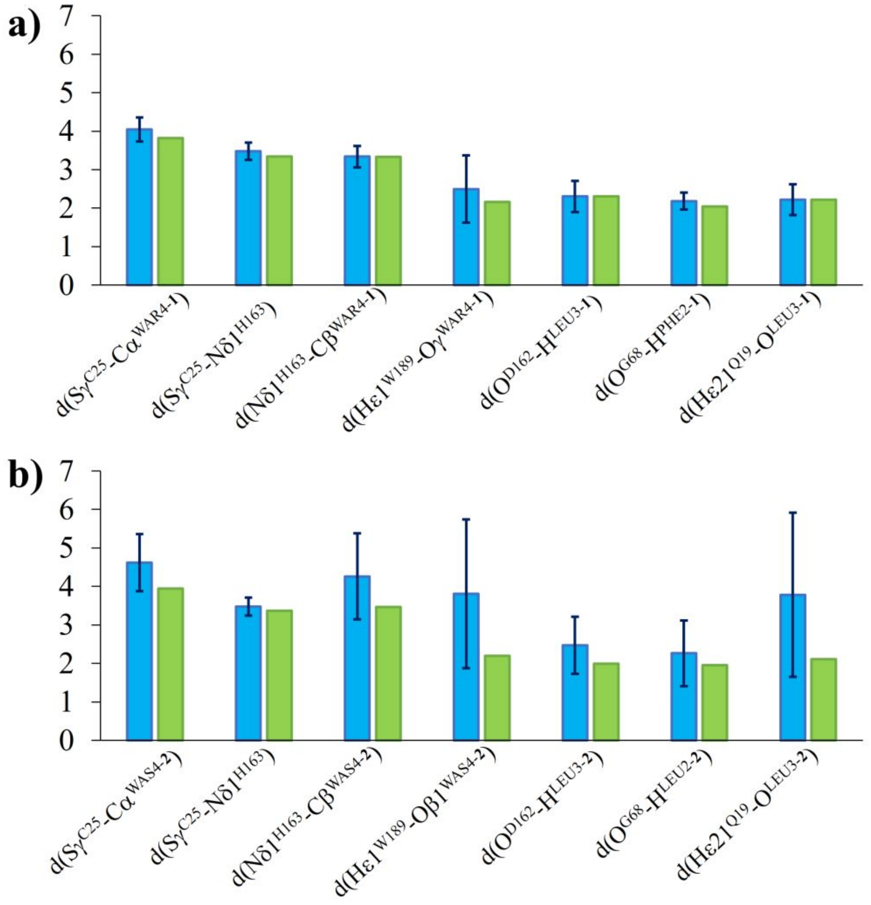

Figure S2. Comparative analysis between the averaged inter-atomic distances computed through the long MM MD simulations of the non-covalent reactant complex (in blue) and the selected final structure employed for the QM/MM calculations (in green) for **a)** compound **1**; and **b)** compound **2**.

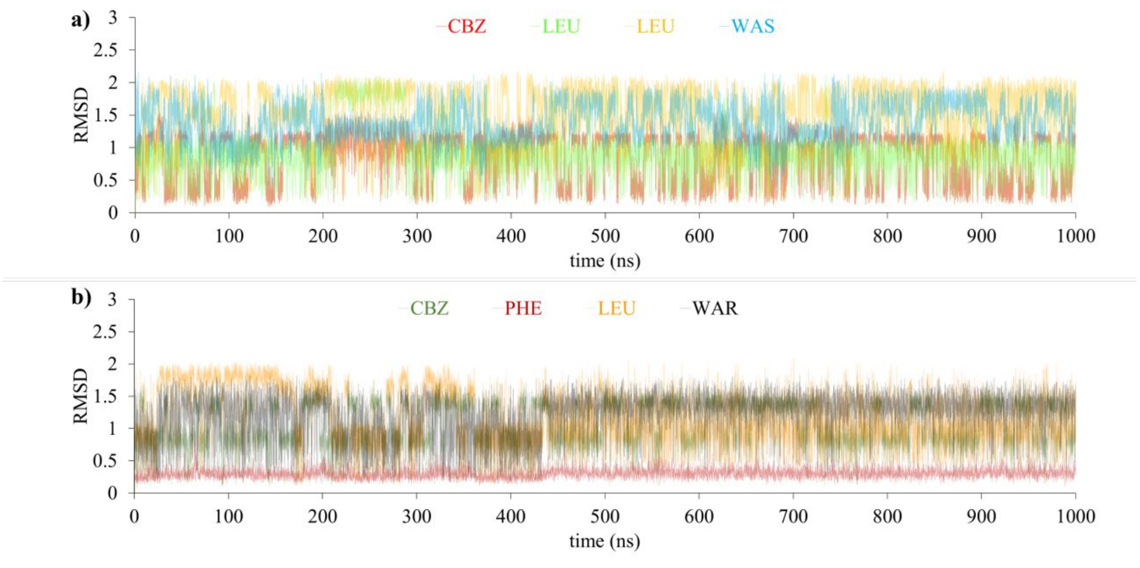

Figure S3. Time evolution of the RMSD (in Å) computed for different parts of the inhibitor separately of **a)** compound **1** and **b)** compound **2** .

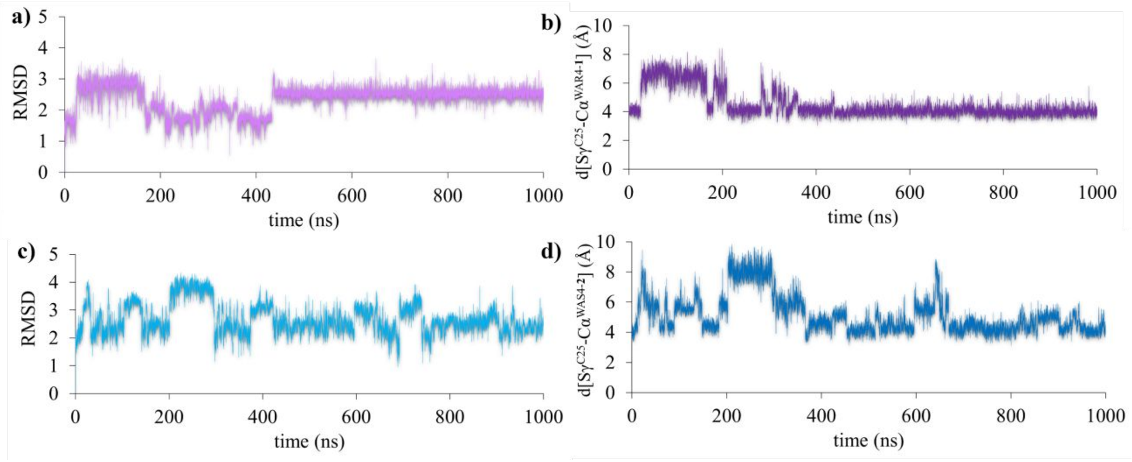

Figure S4. Analysis of structures generated during 1μs MD simulations **a)** RMSD of the position of all atoms of compound **1** **b)** Evolution of the distance between the Sγ sulfur of the catalytic cysteine and the Cα carbon of the double bond of compound **1** **c)** RMSD of the position of all atoms of compound **2** along the MD **d)** Distance between the sulfur of the reactive cysteine and the Cα carbon of the double bond of compound **2**. All RMSD and distances are in Å.

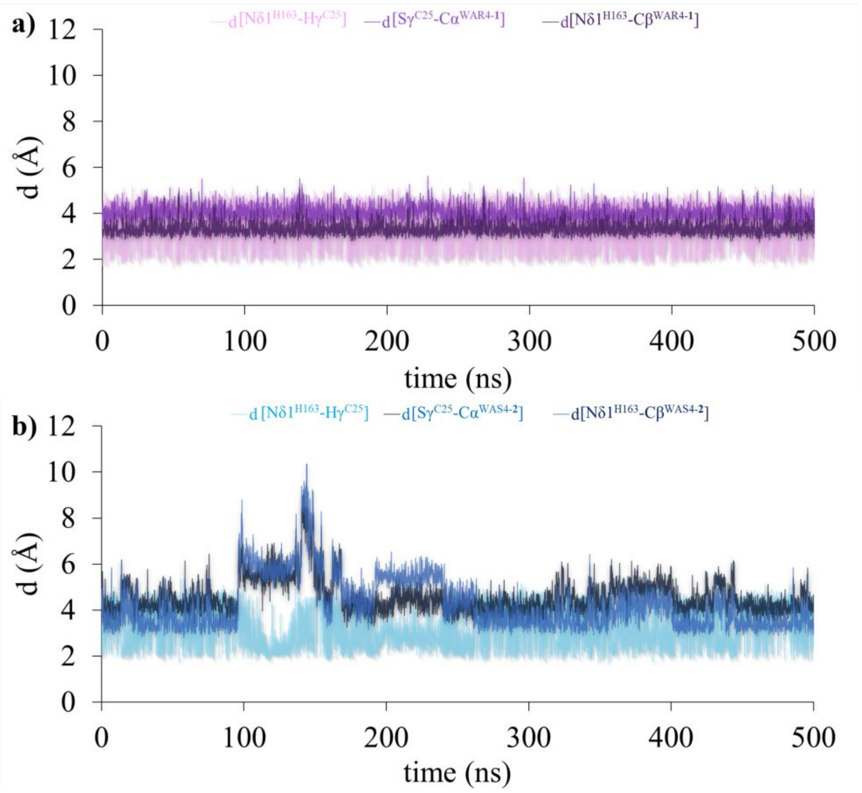

Figure S5. Key distances evolution (in Å) along the last 500 ps of the classical MD for **a)** compound **1;** and compound **2** **b)**.

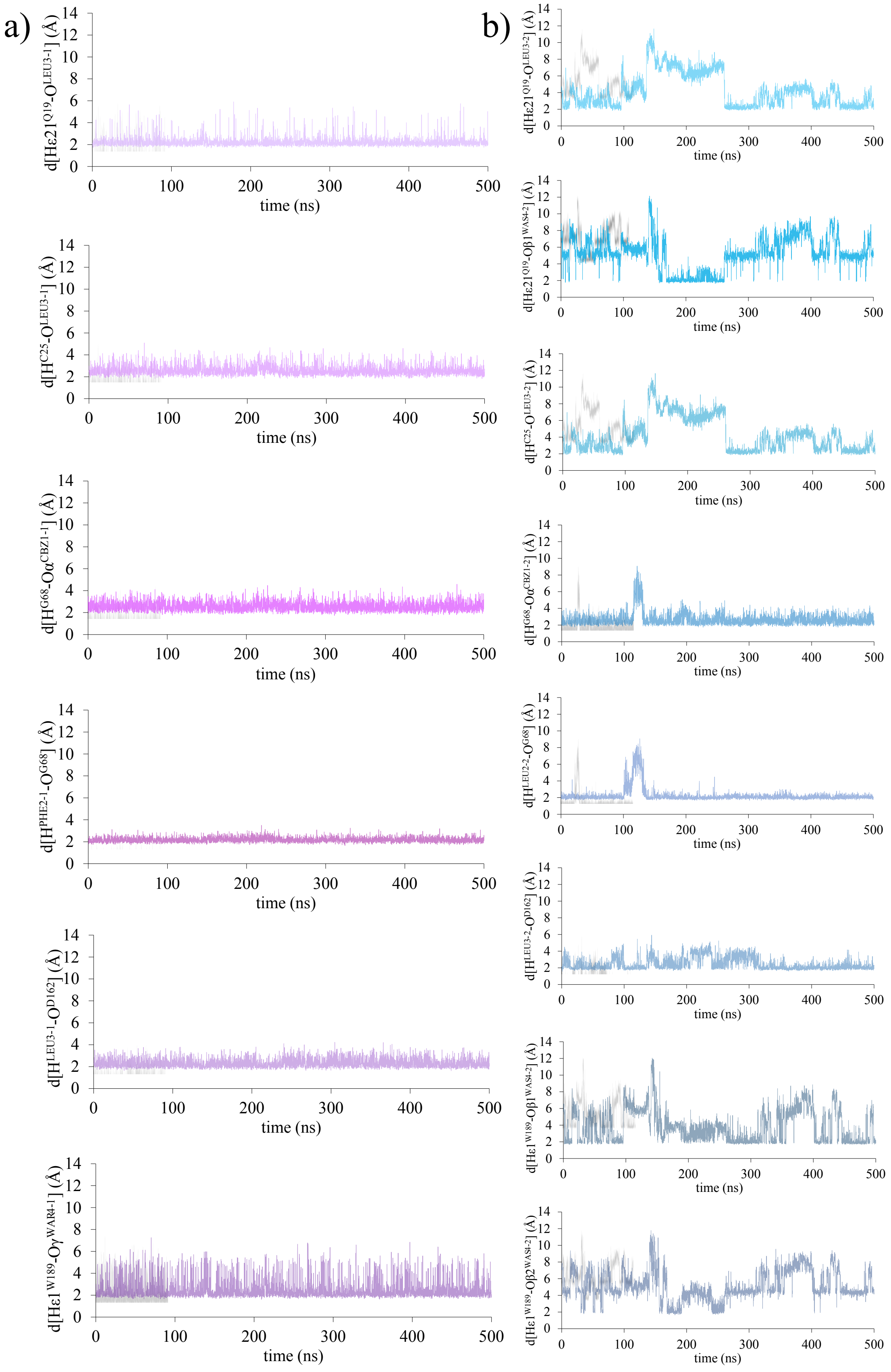

Figure S6. Distance evolution of the most relevant hydrogen bond contacts along the last 500 ps of the classical MD for compound **a) 1 b) 2**.

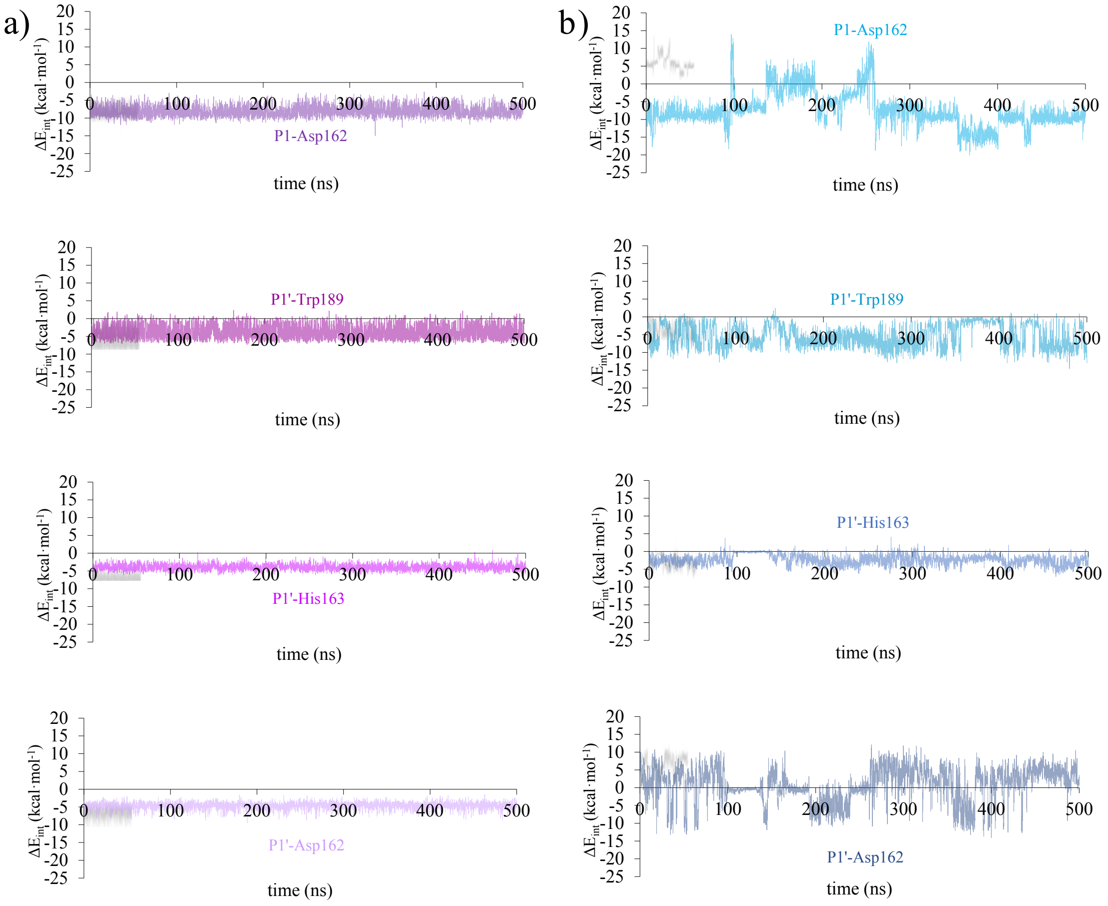

Figure S7. Evolution of most relevant non-bonded interactions between P1 and Asp162 and the warhead and the closest residues along the las 500 ps of the classical MD in compound **a) 1 b) 2**.

Results of QM/MM MD simulations

General comments:

The 2D PESs computed at the AM1/MM level were corrected by means of an energy function to reduce the errors associated with the initial semiempirical level of theory.[1-4] The new energies were computed according to **eq. S1**:

$$E = E_{LL/MM} + S[\Delta E_{LL}^{HL}(\xi_1,\xi_2)] \quad \textbf{(S1)}$$

In this, the final energy is calculated by adding a correction term evaluated from the energy difference between the single points computed at the low level (LL), AM1 in our case, and at the high level (HL), i.e. M06-2X, of theory for the QM region. The basis set employed was 6-31+G(d,p) and the Gaussian09 in combination with fDynamo were the software chosen to perform these PESs.

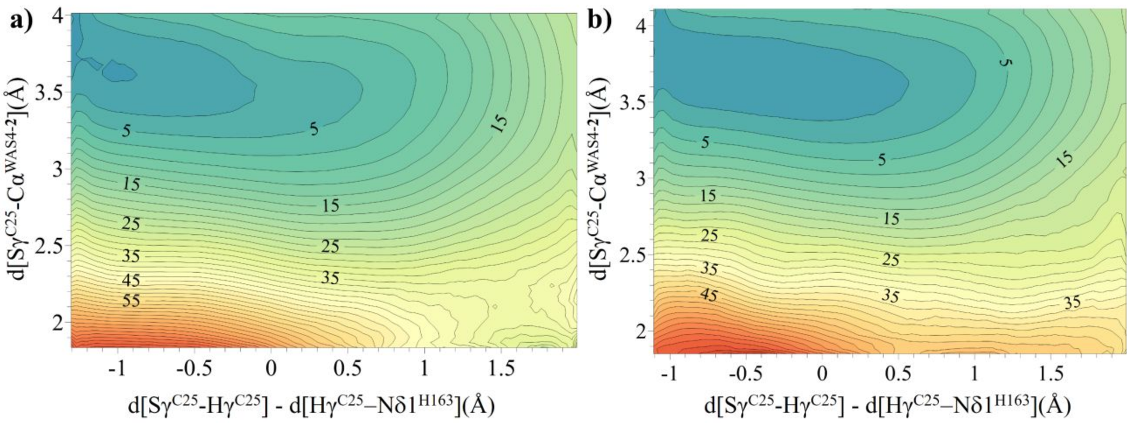

Figure S8. DFT:AM1/MM Potential energy surfaces of the attack of the $S\gamma^{C25}$ sulfur atom to Cα atom of the double bond and the antisymmetric combination control of the key distances for the proton transfer from Cys25 to His163 of **a)** compound **1**; **b)** compound **2**.

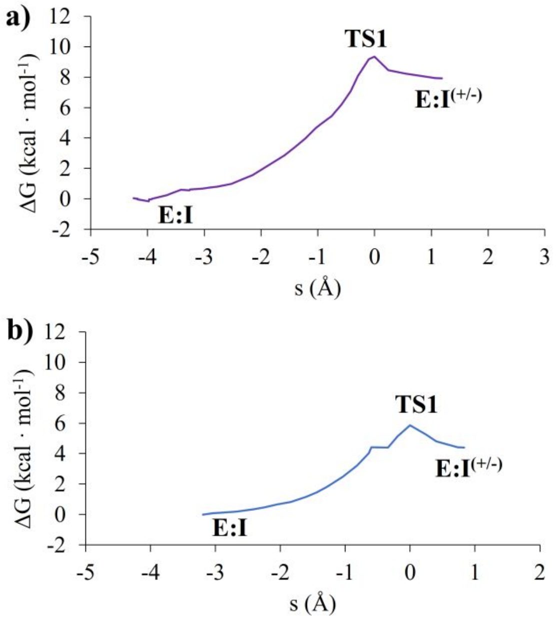

Figure S9. DFT/MM Free energy profiles for the formation of the activated ion-pair, E:I$^{(+/-)}$, from the non-covalent complex E:I, computed by means of the free energy perturbation method at M06-2X/MM level of theory for compound **1** (**a)** and compound **2** (**b**).

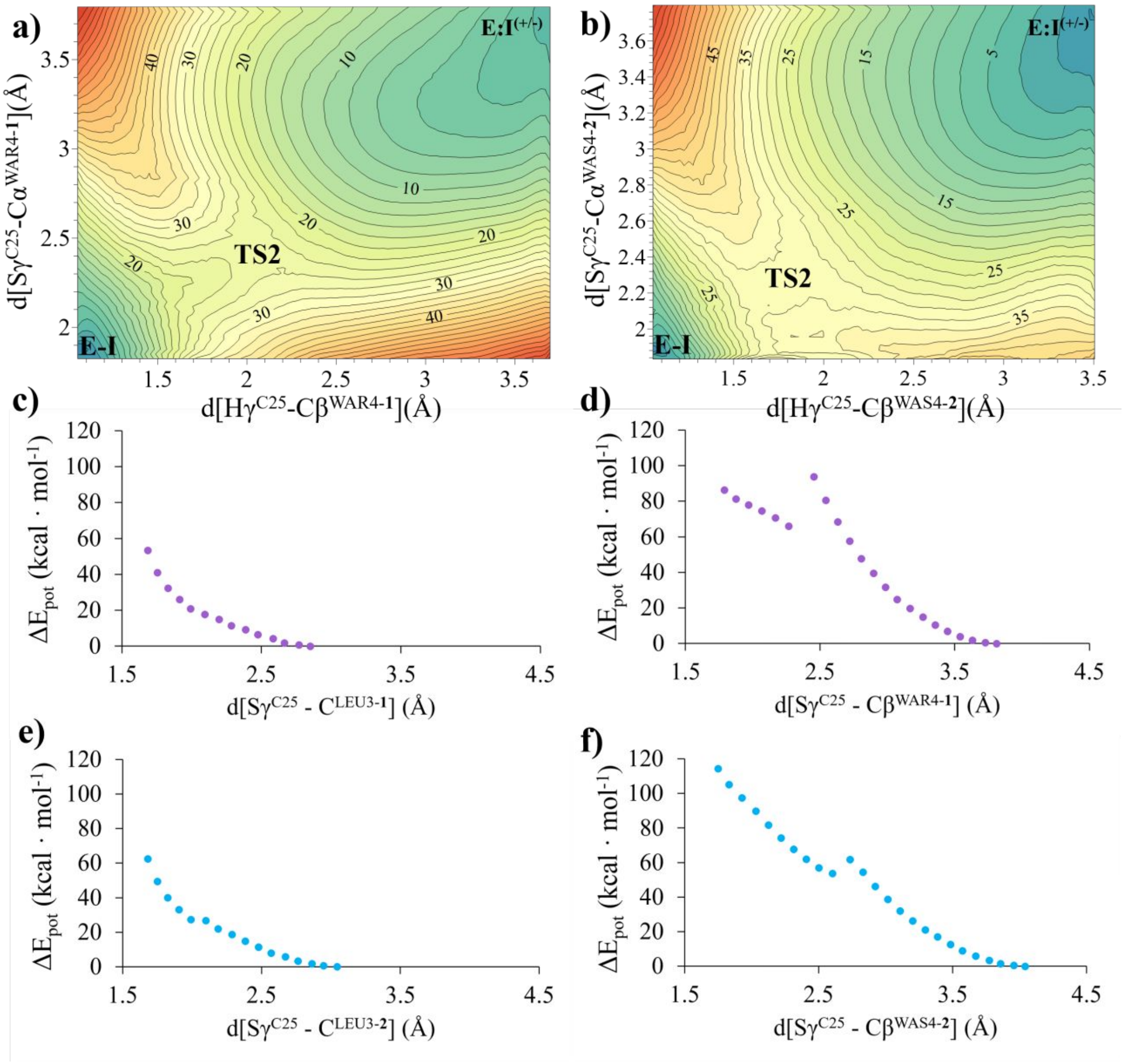

Figure S10. QM/MM Potential Energy Surfaces, PESs, of the attack of the $S\gamma^{C25}$ sulfur atom to: **a)** Cα atom of the double bond of compound **1**; **b)** Cα atom of the double bond of compound **2**; **c)** Carbonyl of LEU3 of compound **1**; **d)** Cβ atom of the double bond of compound **1**; **e)** Carbonyl of LEU3 of compound; and **2** **f)** Cβ atom of the double bond of compound **2**. PESs on panels a and b were computed at DFT:AM1/MM level, while those on panels c-f were computed at DFT/MM level.

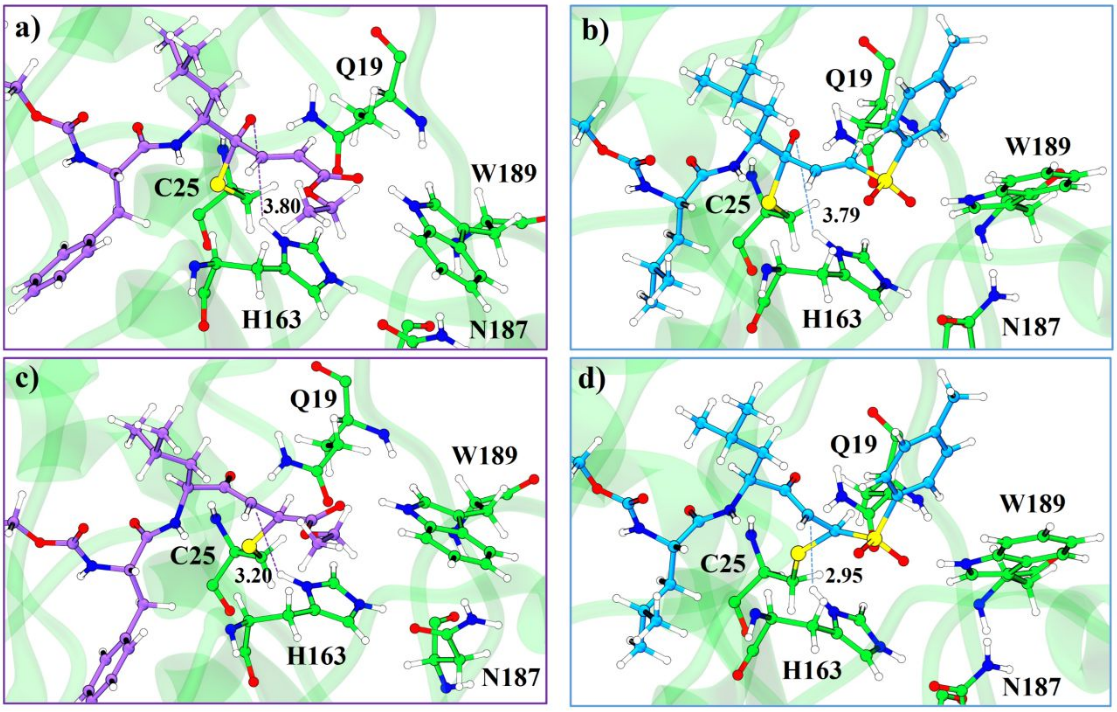

Figure S11. Detail of the active site after the attack of the Sγ^{C25} sulfur atom to **a)** Carbonyl of LEU3 of compound **1** **b)** Cβ atom of the double bond of compound **1** **c)** Carbonyl of LEU3 of compound **2** **d)** Cβ atom of the double bond of compound **2**. Structures optimized at M06-2X/MM level.

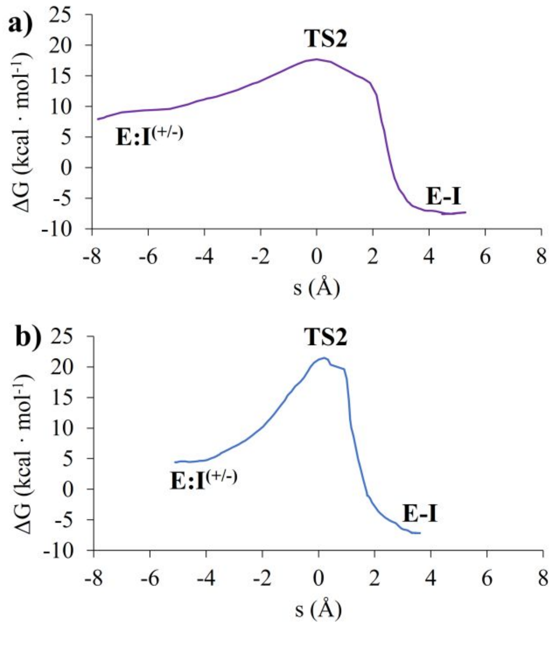

Figure S12. DFT/MM Free energy profiles for the Michael addition reaction from the ion pair E:I$^{(+/-)}$ computed by means of the free energy perturbation method at M06-2X/MM level of theory for compound **1** (**a**) and compound **2** (**b**).

Table S3. Key interatomic distances for all the states appearing along the Michael addition mechanism for compound **1**. All states have been optimized at M06-2X/MM level of theory and the distances are given in Å.

	Compound 1					
	**E:I**	**TS1**	**E:I$^{(+/-)}$**	**E:I$^{(+/-)}$**	**TS2**	**E-I**
H^{C25} - $O^{LEU3\text{-}\mathbf{1}}$	2.37	2.34	2.33	2.31	2.11	2.35
$H\varepsilon 21^{Q19}$ - $O^{LEU3\text{-}\mathbf{1}}$	2.01	1.94	1.92	1.92	1.98	2.04
$H\varepsilon 1^{W189}$ - $O\gamma^{WAR4\text{-}\mathbf{1}}$	1.90	1.94	1.94	1.94	1.91	1.91
$C\alpha^{WAR4\text{-}\mathbf{1}}$ - $C\beta^{WAR4\text{-}\mathbf{1}}$	1.33	1.33	1.34	1.34	1.42	1.53
$H\gamma^{C25}$ - $N\delta 1^{H163}$	2.46	1.28	1.10	1.09	1.04	2.34
$S\gamma^{C25}$ - $N\delta 1^{H163}$	3.51	2.93	3.05	3.06	3.40	3.46
$H\gamma^{C25}$ - $S\gamma^{C25}$	1.35	1.67	1.98	2.00	2.73	2.98
$S\gamma^{C25}$ - $C\alpha^{WAR4\text{-}\mathbf{1}}$	3.63	3.05	3.01	3.00	2.26	1.87
$H\gamma^{C25}$ - $C\beta^{WAR4\text{-}\mathbf{1}}$	2.90	3.02	2.95	2.94	1.98	1.09
$N\delta 1^{H163}$ - $C\beta^{WAR4\text{-}\mathbf{1}}$	2.94	3.21	3.22	3.22	2.93	2.81
$H\varepsilon 2^{H163}$ - $O\delta 1^{N187}$	1.90	1.96	1.95	1.95	1.78	1.90

Table S4. Key interatomic distances for all the states appearing along the Michael addition mechanism for compound **2**. All states have been optimized at M06-2X/MM level of theory and the distances are given in Å.

	Compound 2					
	**E:I**	**TS1**	**E:I$^{(+/-)}$**	**E:I$^{(+/-)}$**	**TS2**	**E-I**
H^{C25} - $O^{LEU3\text{-}\mathbf{2}}$	2.70	2.73	2.70	2.68	2.40	2.83
$H\varepsilon 21^{Q19}$ - $O^{LEU3\text{-}\mathbf{2}}$	1.98	2.01	1.98	1.99	2.03	2.10
$H\varepsilon 1^{W189}$ - $O\beta 1^{WAS4\text{-}\mathbf{2}}$	1.83	1.84	1.83	1.83	1.83	1.85
$C\alpha^{WAS4\text{-}\mathbf{2}}$ - $C\beta^{WAS4\text{-}\mathbf{2}}$	1.33	1.33	1.33	1.33	1.43	1.53
$H\gamma^{C25}$ - $N\delta 1^{H163}$	2.30	1.30	1.11	1.10	1.05	2.34
$S\gamma^{C25}$ - $N\delta 1^{H163}$	3.36	2.92	3.00	3.01	3.27	3.20
$H\gamma^{C25}$ - $S\gamma^{C25}$	1.35	1.65	1.93	1.94	2.68	3.06
$S\gamma^{C25}$ - $C\alpha^{WAS4\text{-}\mathbf{2}}$	3.81	3.26	3.20	3.19	2.19	1.89
$H\gamma^{C25}$ - $C\beta^{WAS4\text{-}\mathbf{2}}$	3.33	3.19	3.13	3.11	1.85	1.09
$N\delta 1^{H163}$ - $C\beta^{WAS4\text{-}\mathbf{2}}$	3.28	3.32	3.35	3.34	2.85	2.96
$H\varepsilon 2^{H163}$ - $O\delta 1^{N187}$	1.95	1.91	1.89	1.88	1.83	1.92

Table S5. Charges in a.u of the most relevant heavy atoms along the mechanism of reaction computed at the M06-2X/MM level of theory for compound **1**.

	Compound 1					
	**E:I**	**TS1**	**E:I$^{(+/-)}$**	**E:I$^{(+/-)}$**	**TS2**	**E-I**
$C\alpha^{WAR4-1}$	-0.27	-0.16	-0.10	-0.10	-0.10	0.01
$C\beta^{WAR4-1}$	-0.24	-0.38	-0.43	-0.43	-0.65	-0.48
$N\delta1^{H163}$	-0.19	0.01	0.13	0.12	0.03	-0.28
$S\gamma^{C25}$	-0.50	-0.80	-0.86	-0.86	-0.68	-0.51
$H\gamma^{C25}$	0.09	0.11	0.04	0.04	0.18	0.19

Table S6. Charges in a.u of the most relevant heavy atoms along the mechanism of reaction computed at the M06-2X/MM level of theory for compound **2**.

	Compound 2					
	**E:I**	**TS1**	**E:I$^{(+/-)}$**	**E:I$^{(+/-)}$**	**TS2**	**E-I**
$C\alpha^{WAS4-2}$	-0.20	0.00	0.05	0.03	0.21	0.15
$C\beta^{WAS4-2}$	-0.34	-0.45	-0.47	-0.46	-0.96	-0.48
$N\delta1^{H163}$	-0.18	0.26	0.39	0.40	-0.18	-0.46
$S\gamma^{C25}$	-0.39	-0.69	-0.78	-0.78	-0.68	-0.47
$H\gamma^{C25}$	0.01	-0.06	-0.14	-0.14	0.41	0.24

References

(1) Ruiz-Pernía J. J.; Silla E.; Tuñón I.; Martí S.; Moliner V. Hybrid QM/MM Potential of Mean Force with Interpolated Corrections. *J Phys Chem* B, **2004**, *108*, 8427-8433. https://doi.org/10.1021/jp049633g

(2) Ruiz-Pernía, J. J.; Silla, E.; Tuñón, I.; Martí, S. Hybrid Quantum Mechanics/Molecular Mechanics Simulations with Two-Dimensional Interpolated Corrections: Application to Enzymatic Processes. *J Phys Chem B* **2006**, 110, 17663−17670. https://doi.org/10.1021/jp063520a.

(3) Chuang, Y.-Y.; Corchado, J. C.; Truhlar, D. G. Mapped Interpolation Scheme for Single-Point Energy Corrections in Reaction Rate Calculations and a Critical Evaluation of Dual-Level Reaction Path Dynamics Methods. *J Phys Chem A* **1999**, 103, 1140−1149. https://doi.org/10.1021/jp9842493.

(4) Świderek, K.; Tuñón, I.; Martí, S.; Moliner, V. Protein conformational landscapes and catalysis. Influence of active site conformations in the reaction catalyzed by L-lactate dehydrogenase. *ACS Catal* **2015**, 5, 1172−1185. https://doi.org/10.1021/cs501704f.
